# Abscisic acid influences tillering by modulation of strigolactones in barley

**DOI:** 10.1093/jxb/ery200

**Published:** 2018-07-05

**Authors:** Hongwen Wang, Wanxin Chen, Kai Eggert, Tatsiana Charnikhova, Harro Bouwmeester, Patrick Schweizer, Mohammad R Hajirezaei, Christiane Seiler, Nese Sreenivasulu, Nicolaus von Wirén, Markus Kuhlmann

**Affiliations:** 1 Department of Physiology and Cell Biology, Leibniz Institute of Plant Genetics and Crop Plant Research (IPK) Gatersleben, Stadt Seeland, Germany; 2 Department of Breeding Research, Leibniz Institute of Plant Genetics and Crop Plant Research (IPK) Gatersleben, Stadt Seeland, Germany; 3 Laboratory of Plant Physiology, Wageningen University, Wageningen, The Netherlands; 4 Plant Hormone Biology Group, Swammerdam Institute for Life Sciences, University of Amsterdam, XH Amsterdam, The Netherlands; 5 Department of Molecular Genetics, Leibniz Institute of Plant Genetics and Crop Plant Research (IPK) Gatersleben, Stadt Seeland, Germany; 6 International Rice Research Institute (IRRI), Grain Quality and Nutrition Center, Metro Manila, Philippines

**Keywords:** Abscisic acid, barley, cereals, hormone regulation, phytohormone cross-talk, shoot branching, strigolactone biosynthesis, tillering

## Abstract

Strigolactones (SLs) represent a class of plant hormones that are involved in inhibiting shoot branching and in promoting abiotic stress responses. There is evidence that the biosynthetic pathways of SLs and abscisic acid (ABA) are functionally connected. However, little is known about the mechanisms underlying the interaction of SLs and ABA, and the relevance of this interaction for shoot architecture. Based on sequence homology, four genes (*HvD27*, *HvMAX1*, *HvCCD7*, and *HvCCD8*) involved in SL biosynthesis were identified in barley and functionally verified by complementation of Arabidopsis mutants or by virus-induced gene silencing. To investigate the influence of ABA on SLs, two transgenic lines accumulating ABA as a result of RNAi-mediated down-regulation of *HvABA 8’-hydroxylase 1* and *3* were employed. LC-MS/MS analysis confirmed higher ABA levels in root and stem base tissues in these transgenic lines. Both lines showed enhanced tiller formation and lower concentrations of 5-deoxystrigol in root exudates, which was detected for the first time as a naturally occurring SL in barley. Lower expression levels of *HvD27*, *HvMAX1*, *HvCCD7*, and *HvCCD8* indicated that ABA suppresses SL biosynthesis, leading to enhanced tiller formation in barley.

## Teaser

Repression of ABA degradation in transgenic barley alters the expression of genes involved in strigolactone biosynthesis, revealing cross-talk between ABA and strigolactone that affects the development of tillers.

## Introduction

Strigolactones (SLs) are a group of carotenoid-derived lactones that have been recently identified as regulators of plant growth and development. SLs were initially characterized as rhizosphere signals that stimulate seed germination in root parasitic plants (Xie *et al.*, 2010; Ruyter-Spira *et al.*, 2013). Later, they were also identified as signals for the establishment of symbiosis with arbuscular-mycorrhizal fungi, a process that improves phosphorus nutrition and water uptake in plants (Akiyama and Hayashi, 2006; Xie *et al.*, 2010). Another major function of SLs is based on their translocation from roots to shoots, where they suppress lateral shoot branching by inhibiting the outgrowth of axillary buds (Gomez-Roldan *et al.*, 2008; Umehara *et al.*, 2008). Insights into the key components of SL biosynthesis and signaling have been gained from studies of mutants with different branching phenotypes in a variety of plant species, such as *ramosus* (*rms*) in pea, *decreased apical meristem* (*dad*) in *Petunia*, *more axillary growth* (*max*) in Arabidopsis, and *dwarf* (*d*)/*high tillering dwarf* (*htd*) in rice (Sorefan *et al.*, 2003; Booker *et al.*, 2005; Johnson *et al.*, 2006; Zou *et al.*, 2006; Arite *et al.*, 2007; Simons *et al.*, 2007; Drummond *et al.*, 2009; Brewer *et al.*, 2016). These studies confirmed a conserved role for SLs in the regulation of axillary bud outgrowth (Domagalska and Leyser, 2011; Ruyter-Spira *et al.*, 2013). SLs are also involved in other aspects of development, including root architecture, leaf senescence, secondary growth, and drought tolerance (Woo *et al.*, 2001; Snowden *et al.*, 2005; Agusti *et al.*, 2011; Kapulnik *et al.*, 2011; Ruyter-Spira *et al.*, 2011; Rasmussen *et al.*, 2012; Bu *et al.*, 2014; Yamada *et al.*, 2014; Ueda and Kusaba, 2015).

**Fig. 1.**
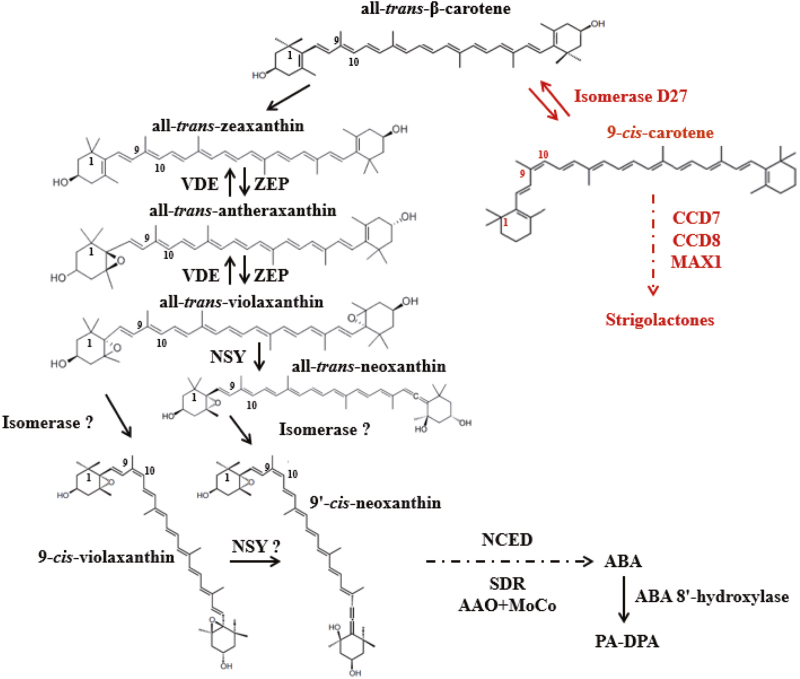
Biosynthetic pathways of abscisic acid and strigolactones. Solid arrows indicate one-step modification of an intermediate and dashed arrows represent multistep modifications of an intermediate. Enzyme names are given in bold. Abbreviations: ZEP, zeaxanthin epoxidase; VDE, violaxanthin de-epoxidase; NSY, neoxanthin synthase; NCED, 9-*cis*-epoxycarotenoid dioxygenase; SDR, short-chain dehydrogenase/reductase; AAO, ABA aldehyde oxidase; *MoCo*, molybdenum cofactor synthase; D27, dwarf 27; CCD7/8, carotenoid cleavage dioxygenase 7/8; MAX1, more axillary growth 1.

To date, more than 20SLs have been isolated and characterized from root exudates of various plant species. All canonical SLs contain a tricyclic lactone (ABC part) that connects via an enol ether linkage to a butenolide moiety (D-ring); they often carry different substituents on the A- and B-rings but have the same C–D moiety (Xie *et al.*, 2013). 5-deoxystrigol and 4-deoxyorobanchol are thought to be the common precursors of the canonical strigol- and orobanchol-type SLs (Al-Babili and Bouwmeester, 2015). Strigolactones are derived from the carotenoid biosynthetic pathway, in which all-*trans*-β-carotene acts as the precursor for SL biosynthesis (Fig. 1). Several enzymes act sequentially in SL biosynthesis (Saeed *et al.*, 2017): DWARF27 (D27) is a β-carotene isomerase, CAROTENOID CLEAVAGE DIOXYGENASE 7 (CCD7) and CCD8 sequentially cleave 9-*cis*-β-carotene to produce the carotenoid precursor carlactone (CL). In rice, CL is oxidized by two cytochrome P450 enzymes (carlactone oxidase and 4-deoxyorobanchol-4-hydroxylase), converting CL into 4-deoxyorobanchol and further into orobanchol (Zhang *et al.*, 2015). MORE AXILLARY GROWTH 1 (MAX1) was identified as a factor upstream of these, providing the early catalytic activity, and both redundancy and functional diversification are associated with gene duplication in angiosperm lineages (Challis *et al.*, 2013). MAX1 was identified as a class Ш cytochrome P450 enzyme in Arabidopsis (Waldie *et al.*, 2014) that converts CL into carlactonoic acid (CLA), which is further converted in a species-specific way to either methyl carlactonoate (MeCLA) (Abe *et al.*, 2014), 4-deoxyorobanchol, or orobanchol (Yoneyama *et al.*, 2018). Finally, MeCLA is the substrate for LATERAL BRANCHING OXIDOREDUCTASE (LBO), resulting in the formation of an unidentified SL-like compound (Brewer *et al.*, 2016). This core set of enzymes is mostly active in roots, and root-synthesized SLs are secreted by ABCG-type transporters such as PhPDR1 into the rhizosphere or transported to the shoot (Kretzschmar *et al.*, 2012; Sasse *et al.*, 2015). Perception and signaling of SLs in plants requires two major proteins, namely MAX2, an F-box protein of the Skp–cullin–F-box (SCF) E3 ubiquitin ligase complex (Stirnberg *et al.*, 2007; Smith and Li, 2014), and DWARF14 (D14), an α/β-fold hydrolase protein (Arite *et al.*, 2009; Hamiaux *et al.*, 2012). The D14 protein has been shown to act as a SL receptor by hydrolysing SLs into a covalently linked intermediate, which initiates a conformational change of D14 that facilitates its interaction with MAX2 (de Saint Germain *et al.*, 2016; Yao *et al.*, 2016). In barley, the identification and functional analysis of D14 was achieved by analysis of tilling mutants (Marzec *et al.*, 2016).

Abscisic acid (ABA) is a key regulator of plant responses to abiotic stresses and is involved in numerous developmental processes (Vishwakarma *et al.*, 2017). Both ABA and SLs are carotenoid-derived molecules (apocarotenoids) that share all-*trans*-β-carotene as a common biosynthetic precursor (Matusova *et al.*, 2005). The precursor is derived from the isoprene derivative isopentenyl diphosphate (IPP) (Schwartz *et al.*, 1997) and is converted by a series of epoxidation and hydroxylation reactions into two all-*trans*-epoxycarotenoids, namely all-*trans*-violaxanthin and all-*trans*-neoxanthin (Al-Babili and Bouwmeester, 2015). Violaxanthin and neoxanthin are then converted into 9-*cis* and 9’-*cis* isomers, respectively, by an unknown *cis*/*trans* isomerase. Alder *et al.* (2012) found that the rice SL biosynthetic enzyme D27 has isomerase activity, converting all-*trans*-β-carotene into 9-*cis*-β-carotene, and that this conversion is reversible. The structural similarity between β-carotene and epoxycarotenoids raises the possible involvement of D27 in ABA biosynthesis. Next, 9-*cis*-EPOXYCAROTENOID DIOXYGENASE (NCED) catalyses the oxidative cleavage of 9-*cis*-violaxanthin and/or 9’-*cis*-neoxanthin to produce xanthoxin, which is considered the rate-limiting step in ABA biosynthesis (Chernys and Zeevaart, 2000; Iuchi *et al.*, 2001; Qin and Zeevaart, 2002). For ABA catabolism, the majority of ABA is converted to its inactive form via hydroxylation at the 8’-carbon atom, and this is carried out by the cytochrome P450-type protein ABA-8’-HYDROXYLASE. In barley, three members of the *ABA8’-OH* gene family have been described (Seiler *et al.*, 2014). Spontaneous cyclization of hydroxylated ABA results in the formation of phaseic acid (PA), which is further reduced to dihydrophaseic acid (DPA) by a soluble reductase. The spontaneous cyclization to PA results in a significant reduction in the biological activity, and reduction to DPA results in a complete loss of activity (Zou *et al.*, 1995; Zhou *et al.*, 2004; Seiler *et al.*, 2011). In line with its important role as a phytohormone, a fine-tuned balance between biosynthesis, inactivation, and degradation controls ABA homeostasis (Seiler *et al.*, 2011).

Given its biosynthetic origin and the importance of ABA in abiotic stress responses, attention has been drawn to a possible interaction between it and SLs, and the consequences this would have for abiotic stress responses. In drought-stressed leaves, ABA levels usually rise due to increased NCED activity, which results in stomatal closure (Zhu, 2002). In *Lotus japonicus*, osmotic stress was found to decrease SL levels in root tissues and exudates, primarily by altering transcript levels of biosynthetic and transporter-encoding genes (Liu *et al.*, 2015). Pre-treatment of plants with SLs inhibited the osmotic stress-induced ABA increase in roots by down-regulating the ABA biosynthetic gene *LjNCED2*. During osmotic stress in *L. japonicus* roots, only a decrease in SL levels allowed ABA to increase (Liu *et al.*, 2015). A negative interaction between ABA and SLs was also observed in tomato (Ruiz-Lozano *et al.*, 2016). In Arabidopsis mutants, a suppressive function of ABA was shown for axillary bud outgrowth that depended on the ratio of red to far-red light (Reddy *et al.*, 2013; Yao and Finlayson, 2015). However, the molecular mechanism underlying the interaction of SLs and ABA remains unclear.

In this study, we investigated the interaction between ABA and SLs and its effect on shoot architecture in barley under non-stressed conditions. Employing *HvABA 8’-hydroxylase* RNAi plants (Seiler *et al.*, 2014), we analysed phytohormone levels and tiller formation. As a prerequisite for this study, four barley genes involved in SL biosynthesis were identified and functionally characterized by genetic complementation and virus-induced gene silencing (VIGS). Our results indicate that enhanced levels of endogenous ABA cause a decrease in SL production via repression of SL biosynthesis genes during a late developmental phase in barley. Our results provide genetic evidence for an antagonistic interaction between SLs and ABA that takes place at the level of SL biosynthesis.

## Materials and methods

### Plant material and cultivation

*Hordeum vulgare* cv. Golden Promise (two-rowed spring barley cultivar, from the IPK Gatersleben gene bank) and the derived transgenic lines LOHi236 and LOHi272 (Seiler *et al.*, 2014) were cultivated in a growth chamber. Seeds were sown directly in pots filled with two parts compost, two parts ‘Substrat 2’ (Klasmann-Deilmann), and one part quartz sand. Vernalization was performed for a period of 8 weeks from germination under a temperature regime of 11/7 °C day/ night with 10 h light. Growing conditions were divided into four phases: (1) 2 weeks at 14 /9 °C day/night with 12 h light; (2) 2 weeks at 16/9 °C day/night with 14 h light; (3) 2 weeks at 20/12 °C day/night with 16 h light; and (4) ongoing 20/14 °C day/night with 16 h light. The plants were fertilized regularly with Plantacote pluss (15 g pot^-1^; Aglukon) during the vegetative phase and with ‘Hakaphos Rot’ liquid fertilizer (once a week; 2–4%; Compo Expert) from the start of spike development. Total tiller number until maturity was counted for all plants every week (*n*≥20 plants). *Hordeum vulgare* cv. Black Hulless, a six-rowed spring barley cultivar, was used for the VIGS experiments (Scofield *et al.*, 2005)) and grown in a climate chamber at 20–25 °C with a 16/8 h light/dark photoperiod until the two-leaf stage, then inoculated with Barley stripe mosaic virus (BSMV), kept for 2 weeks and transferred to a growth chamber (22/18 °C day/night with 16 h light).

*Arabidopsis thaliana* Columbia-0 (Col-0), the *Atd27* mutant (GABI-Kat collection, GK-134E08.01) (Waters *et al.*, 2012), and the *Atmax1-1* mutant, kindly provided by Prof. Ottoline Leyser (Booker *et al.*, 2005) were used for genetic complementation experiments. Arabidopsis seeds were surface-sterilized with 20% (v/v) bleach for 15 min, washed, and plated on Murashige and Skoog (MS) medium supplemented with 2% (w/v) sucrose for 10 d. Seedlings with the same size were transferred to soil under a 16/8 h light/dark, 22/18 °C long-day (LD) regime with a light intensity of 120–150 μmol m^−2^ s^−1^. Pots were placed in the growth room in a randomized design. Observations of primary shoot branching phenotype were repeated at least two times (*n*=15–20).

### Phylogenetic analyses

Amino acid sequence alignments were performed using the ClustalW module in the MEGA 6.0 program (Tamura *et al.*, 2013). Neighbor-joining trees and bootstrap analyses were also conducted using MEGA 6.0, and the following parameters were selected: model, Jones-Taylor-Thornton (JTT); bootstrap, 1000 replicates; and gap/missing data, pairwise deletion. The protein sequences utilized in this analysis as well as NCBI accession numbers and data sources are listed in Supplementary Table S3 at *JXB* online.

### Generation of the BSMV-*HvCCD7* and BSMV-*HvCCD8* constructs and BSMV-mediated virus-induced gene silencing

*HvCCD7* sequences (295 bp and 306 bp) were amplified using *Pfu* DNA polymerase (Thermo Scientific) with gene-specific primers harboring the *BamHI* and *PacI* restriction sites (see Supplementary Table S1), and then directionally cloned in antisense orientation into the *BamHI*- and *PacI*-digested BSMV-γ-MCS multiple cloning site (Bruun-Rasmussen *et al.*, 2007) to generate BSMV-*HvCCD7*-1 and BSMV-*HvCCD7*-2, respectively (Supplementary Fig. S1a). Using the same strategy, the constructs BSMV-*HvCCD8*-1 (202 bp) and BSMV-*HvCCD8*-2 (338 bp) were also generated (Fig. S1b; Table S1). Virus-induced gene silencing (VIGS) was performed according to Bruun-Rasmussen *et al.* (2007). BSMV sub-genomic components α, β, and γ were linearized by *Mlu*I digestion (T7-α and T7-γ recombinant) or *Spe*I (T7-β) and used as templates for *in vitro* transcription using the AmpliCap-Max™ T7 High Yield Message Maker Kit (CELLSCRIPT™), following the manufacturer’s protocol. The three viral RNA components were mixed in a 1:1:1 ratio and 4.5 μl of combined transcripts were mixed with 10 μl of FES buffer (77 mM glycine, 60 mM K_2_HPO_4_, 22 mM Na_4_P_2_O_7_.10H_2_O, and 1% w/v bentonite) and applied with gentle strokes to Bentonite-dusted leaves ((Holzberg *et al.*, 2002). Only plants showing virus symptoms 14 d post-inoculation (dpi) were included in further analysis. Presence of the virus was further confirmed by RT-PCR at 21 dpi. Experiments were repeated three times and *n*=12 plants per construct were evaluated for tiller number.

### Vector constructions of *pAtD27::HvD27* and *35S::HvMAX1*

The *AtD27* promoter was amplified from genomic DNA and ligated into the pCR™2.1-TOPO^®^ vector (Invitrogen). Using *SpeІ* and *XhoІ*, the sequence was further constructed into the *pd35S*-Nos-AB-M vector (see Supplementary Fig. S14). The plasmid sequence *pAtD27*-Nos-AB-M was confirmed by digestion and sequencing. For sub-cloning of *HvD27* under the control of the *AtD27* promoter, the sequence was amplified from barley leaf cDNA and transferred into the TOPO-TA vector and confirmed by sequencing. The *HvD27* sequence was amplified with gene-specific primers having the *XhoІ* and *SalІ* sites. To generate *pAtD27::HvD27*-Nos-AB-M, the PCR product was ligated via *XhoІ* and *SalІ* into *pAtD27*-Nos-AB-M. The *pAtD27::HvD27*-Nos-AB-M cassette was then transferred into the p6U10 binary vector for plant transformation (Supplementary Fig. S14).

For construction of the *pd35S::HvMAX1*-Nos-AB-M overexpression cassette, *HvMAX1* was amplified from barley leaf cDNA and transferred into the pCR™2.1-TOPO^®^ vector (Invitrogen). The plasmid sequence was confirmed by sequencing. The *HvMAX1* TOPO clone was amplified with gene-specific primers having the *XhoІ* and *HindIII* sites. To generate *pd35S::HvMAX1*-Nos-AB-M, the PCR product was ligated via *XhoІ* and *HindIII* to *pd35S*-Nos-AB-M. The *pd35S::HvMAX1*-Nos-AB-M was then transferred into the p6U10 binary vector for plant transformation. The primers used are listed in Supplementary Table S1. The vectors were transformed into *Agrobacterium tumefaciens* strain EHA105 (Chetty *et al.*, 2013) and used for floral dip transformation (Clough and Bent, 1998) of Col-0 and the respective mutants. Resistant plants were selected on half-strength MS medium supplemented with 50 μg ml^–1^ hygromycin. Plates were kept at 4 °C for 2 d and then transferred to growth chamber conditions. Selected plants (T1) were transferred to Arasystem trays (BetaTech bvba, Gent, Belgium) containing Soilrite MixTM. The segregation ratio was estimated from T2 plants. Ten lines (T2) showing a 3:1 segregation ratio were further selected on antibiotic-containing medium to obtain homozygous lines. Analysis was done on T3 homozygous transgenic lines, and their transgenic status was confirmed by RT-PCR analyses.

### RNA extraction, RT-PCR, and qRT-PCR

Fresh plant tissues from independent or pooled biological replicates (e.g. manually dissected meristems) from the same treatment were ground to fine powder in liquid nitrogen and stored at –80 °C. Total RNA was isolated from 100 mg ground material using TRIZOL reagent (Invitrogen GmbH, Karlsruhe, Germany) and RNAeasy columns (Qiagen, Hilden, Germany), treated with RNAse-free DNase (Ambion), and finally dissolved in 50 μl RNase-free water. After performing an RNA quality test, concentrations were measured using a NanoDrop photometer (Peqlab). cDNA synthesis was carried out using SuperScript^TM^ III (Invitrogen GmbH) with 1 μg of total RNA, following the manufacturer’s instructions. For semi-quantitative RT-PCR, 1 μl of diluted cDNA (1:3) was used in a final PCR volume of 20 μl and run for 35 cycles. PCR products (12 μl) were separated on a 1% (w/v) agarose gel.

For real-time qRT-PCR, the reactions were performed in 384-well plates using an ABI PRISM 7900 HT Sequence Detection System and SYBR Green (Applied Biosystems). The PCR protocol consisted of an initial denaturation step (50 °C for 2 min, 95 °C for 10 min), followed by 45 cycles of 95 °C for 15 s and 60 °C for 60 s. Dissociation curves were recorded and data were analysed using SDS2.2.1 software (Applied Biosystems). CT values for each primer set were normalized to the CT value of the reference amplicon (serine/threonine protein phosphatase PP2A-4, catalytic subunit, EST clone HZ44D03).

PCR efficiency was calculated from the slope of the exponential phase of the amplification, following the suggestions made by Ramakers *et al.* (2003). The changes in relative expression levels of target genes in the qRT-PCR were calculated according to the 2^–ΔΔCT^ method (Livak and Schmittgen, 2001). Primer sequences can be found in Supplementary Table S1.

### Extraction and quantification of SLs and ABA

ABA was extracted from fresh plant material using ethyl acetate (100%). Isotopically labelled D6-ABA was used as an internal standard and added to each sample during the extraction procedure. Extraction was carried out twice with 1 ml of ethyl acetate at 4 °C. The supernatant collected after centrifugation (13.000 *g*, 10 min, 4 °C) was evaporated to dryness at room temperature using a vacuum concentrator. The dried samples were dissolved in acetonitrile:methanol (1:1) and filtered using a 0.8-µm filter (Vivaclear). The filtrate (10 µl) was used for subsequent quantification by LC-MS/MS (Dionex Summit coupled to Varian 1200 L). Chromatographic separation was carried out on a C18 column (4 µm, 100 mm; GENESIS; Vydac/USA). Multiple reaction monitoring (MRM) and quantification were done using the mass traces 263/153 for ABA and 269/159 for D6-ABA. The validity of the extraction and measurement procedure was verified in recovery experiments (approx. 82–95%). Quantification was based on calibration with known ABA standards and individual recovery rates for the samples, as described previously (Kong *et al.*, 2008).

For the collection of root exudates, 10 germinated seeds of each line were planted in a 3-l plastic pots filled with 1.5 l of silver sand. After 1 week, plants were thinned to five plants per pot. Each pot was supplemented with 500 ml of half-strength modified Hoagland’s nutrient solution containing 0.1 mM KH_2_PO_4_ (Supplementary Table S2) at 48-h intervals. The plants were allowed to grow in a growth chamber for 13 weeks, and in the 14th week they were watered with a P-deficient nutrient solution (Hoagland’s nutrient solution with 0 mM KH_2_PO_4_) to increase SL production: 3 l of P-deficient nutrient solution was added to the top of each pot and allowed to drain freely through the holes in the bottom to remove P from the sand. The plants were kept under P-deficiency for 1 week. In the 15th week, the same draining with 3 l of P-deficient nutrient solution was repeated to remove any accumulated SLs. Then, 48 h later, root exudates were collected in a 1-l plastic bottle by passing 1 l of nutrient solution without phosphate through each pot. The root exudates were passed through an SPE C18-fast column (500 mg), and SLs were eluted with 6 ml of 100% acetone. For root extracts, 1 g fresh weight of ground root tissue was extracted following the method described by Jamil *et al.* (2012). The resulting extracts were evaporated to dryness, taken up in hexane, loaded on pre-equilibrated Silica gel Grace Pure SPE (200 mg) columns, and eluted with 2 ml of hexane:ethyl acetate (1:9) for further purification. The solvent was evaporated, and the residue was dissolved in 200 μl of 25% (v/v) acetonitrile in water and filtered through Minisart SRP4 0.45-μm filters (Sartorius) before LC-MS/MS analysis.

SLs were analysed by comparing retention times and mass transitions with SL standards (2’-*epi*-5-deoxystrigol, 5-deoxystrigol, and D6-2’-*epi*-5-deoxystrigol) using a Waters Xevo TQ mass spectrometer equipped with an electrospray-ionization source and coupled to a Waters Acquity Ultraperformance LC system using the settings described by Jamil *et al.* (2012) with some modifications (Cardoso *et al.*, 2014). D6-2’-*epi*-5-deoxystrigol served as an internal standard that was added before analysis. Detection and quantification of SLs were performed with three biological replicates.

### Statistical analysis

Values derived from several biological replicates were used to calculate means (±SD), and statistical significance was assessed using Student’s *t*-test and one-way ANOVA across genotypes with Tukey’s multiple comparison test (*P*˂0.05). These calculations were performed using Genstat, 17th edition (VSN International Ltd, Hemel Hempstead, UK).

## Results

### Identification of barley genes homologous to those involved in strigolactone biosynthesis in Arabidopsis

Despite the importance of SLs, rather limited information is available for this class of plant hormones in barley. In order to identify genes involved in SL biosynthesis in barley, protein sequences from Arabidopsis and rice were used as templates to perform a protein homology search based on sequences derived from the barley genome (International Barley Genome Sequencing Consortium, 2012) (http://webblast.ipk-gatersleben.de/barley/index.php; see Supplementary Fig. S2, Table S3). For D27, two amino acid sequences were identified: BAJ90178.1 (Hordeum_10) with 79.2% similarity to rice OsD27 (Lin *et al.*, 2009), and BAJ85620.1 (Hordeum_16) with 47.2% similarity (Supplementary Table S3). Phylogenetic relationships among protein sequences of orthologous genes were determined by arbitrarily rooted neighbor-joining algorithms (Supplementary Fig. S2a). The phylogenetic reconstitution included confirmed and putative D27 sequences from graminaceous and dicotyledonous species and showed that only BAJ90178.1 grouped closely with OsD27 in a sub-cluster of orthologous proteins. This sub-cluster also contained the D27 orthologues from *Medicago truncatula* (van Zeijl *et al.*, 2015) and Arabidopsis, encoded by AT1G03055 (Waters *et al.*, 2012). Thus, BAJ90178.1 (encoded by MLOC_67450, HORVU7Hr1G096930) was designated as HvD27.

In order to identify the orthologous sequences of CCD7 and CCD8 in barley, the amino acid sequences of AtCCD7 (NP_182026.5 encoded by AT2G44990) and AtCCD8 (NP_195007.2 encoded by AT4G32810) were used as references (Wang *et al.*, 2011). The retrieved barley amino acid sequences encoded by MLOC_55474.1 (HORVU2Hr1G097730) and MLOC_66551.1 (HORVU3Hr1G071170) were most closely related to CCD7 and CCD8, respectively, showing 33.7% and 56.6% identity. Subsequent phylogenetic analysis divided the CCD7 and CCD8 proteins into two distinct clusters (Supplementary Fig. S2b). As MLOC_55474.1 grouped together with OsCCD7 and Arabidopsis MAX3/CCD7, it was designated as HvCCD7. Likewise, MLOC_66551.1 grouped together with OsCCD8 and Arabidopsis MAX4/CCD8. Based on their sequence identity, MLOC_55474.1 and MLOC_66551.1 were designated further as HvCCD7 and HvCCD8, respectively.

A BLAST search using AtMAX1 (NP_565617.2) encoded by AT2G26170 as the target (Abe *et al.*, 2014) yielded two barley sequences. A reconstituted phylogeny including sequences from monocot and dicot plants inferred at least two separate clades, with the second clade harboring only a small number of orthologous sequences from graminaceous species, including BAJ97619.1 (Hordeum_2; Supplementary Fig. S2c). The barley sequence BAJ98237.1 (Hordeum_1, AK367034, encoded by HORVU4Hr1G079620) grouped closely to Arabidopsis MAX1 (57% sequence identity) and was therefore designated as HvMAX1. Based on their homology and phylogenetic relationships, the identified barley genes (see genome map in Supplementary Fig. S2d) were selected for further characterization of their putative functions in the SL biosynthesis pathway.

### Functional characterization of *HvCCD7* and *HvCCD8* genes by virus-induced gene silencing in barley

**Fig. 2.**
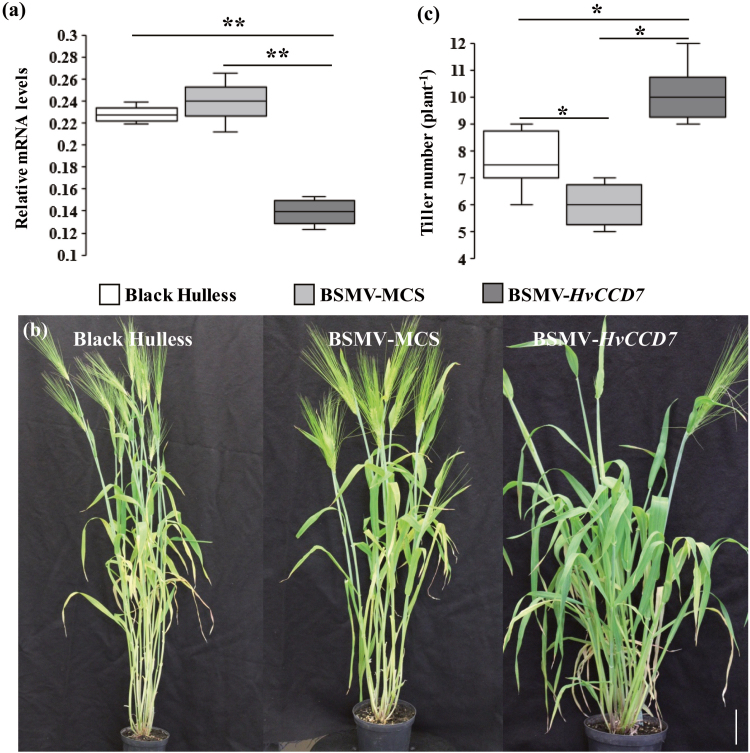
Virus-induced gene silencing (VIGS) of *HvCCD7* in barley. (a) *HvCCD7* transcript levels in leaf tissue as determined by qRT-PCR. *Serine/threonine protein phosphatase PP2A-4* was used as the reference gene. The box-plots represent the results from three independent samples. (b) The tillering phenotypes of BSMV-MCS and BSMV-*HvCCD7*-silenced plants and the reference variety Black Hulless. The scale bar is 10 cm. (c) Total tiller number per plant. The box-plots represent of the results from 12 plants per genotype. Significant differences according to Student’s *t*-test are indicated: *P˂0.05, **P˂0.01.

Gene-silencing using vectors derived from BSMV represents an efficient approach to study gene functions in barley (Pacak *et al.*, 2010). For the functional characterization of *HvCCD7* and *HvCCD8* gene products, we implemented VIGS employing coding sequences that were re-confirmed by sequencing. The VIGS approach was chosen for two reasons: first, the promotor region of the identified genes was still unknown; and second, the expressions of *HvCCD7* and *HvCCD8* were weaker compared to *HvD27* and *HvMAX1* (Supplementary Fig. S7). The coding sequence of each target gene was subjected to an *in silico* analysis using the si-Fi software (siRNA Finder; http://labtools.ipk-gatersleben.de/) to select sequences of 200–340 nucleotides that were predicted to produce a large number of silencing-effective siRNAs. Two VIGS constructs were generated carrying antisense cDNA fragments from either *HvCCD7* or *HvCCD8* (Supplementary Fig. S1). The second leaf of a barley plant was infected with one of these constructs or with the empty BSMV vector (BSMV-MCS). At 9–11 dpi, leaf numbers two and three began to display typical mosaic-type necrosis and curling symptoms (Supplementary Fig. S3a). As confirmed for *HvCCD7* by RT-PCR, harvested leaf material showing these symptoms contained viral DNA (Fig. S3b). The extent of the silencing response of *HvCCD7* was monitored by qRT-PCR on RNA from leaves with the most pronounced symptoms. Relative to non-transformed wild-type plants, BSMV-MCS-infected plants showed a slightly (but not significantly) elevated mRNA level of *HvCCD7*. In contrast, BSMV-*HvCCD7*-infected plants showed a significant reduction in mRNA levels (Fig. 2a). After 15 weeks of growth, the BSMV-MCS-infected plants produced on average one tiller less than non-infected plants (Fig. 2b). BSMV-*HvCCD7*-infected plants were stunted in growth and developed on average three tillers more than the BSMV-infected control (Fig. 2c). Due to their late outgrowth, most tillers in BSMV-*HvCCD7*-silenced plants did not develop ears. This phenotype resembles the *dit1* rice mutant defective in *OsCCD7* (Kulkarni *et al.*, 2014).

**Fig. 3.**
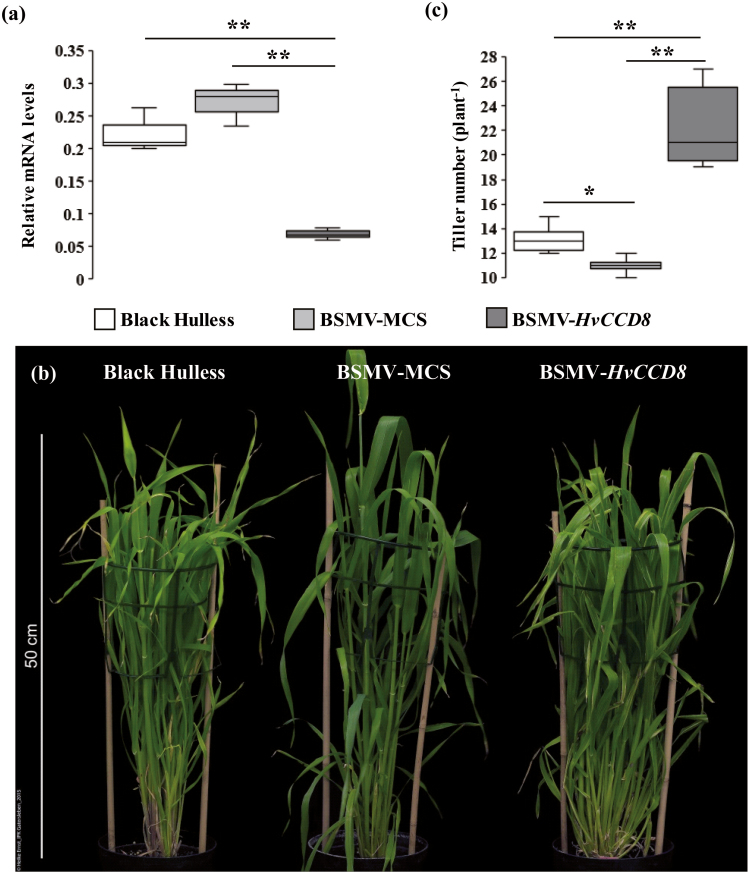
Virus-induced gene silencing (VIGS) of *HvCCD8* in barley. (a) *HvCCD8* transcript levels in leaf tissue as determined by qRT-PCR. *Serine/threonine protein phosphatase PP2A-4* was used as the reference gene. The box-plots represent the results from three independent samples. (b) The tillering phenotype of BSMV-MCS and BSMV-*HvCCD8*-silenced plants and the reference variety Black Hulless. (c) Total tiller number per plant. The box-plots represent of the results from 12 plants per genotype. Significant differences according to Student’s *t*-test are indicated: *P˂0.05, **P˂0.01.

In parallel, barley plants infected with an antisense construct of *HvCCD8* were found to severely suppress mRNA levels of *HvCCD8* (Fig. 3a). These plants showed a bushy phenotype, which was accompanied by continuous tillering (Fig. 3b). After 11 weeks of growth under long-day conditions, these plants had produced twice as many tillers as BSMV-infected control plants (Fig. 3c). This phenotype is in accordance with the expectation of enhanced tiller formation caused by suppressed SL biosynthesis.

### Functional characterization of *HvD27* and *HvMAX1* genes by genetic complementation analysis

In order to confirm a function of *HvD27* and *HvMAX1* in shoot branching, a heterologous complementation approach was employed. The Arabidopsis mutant *d27* (Waters *et al.*, 2012) and Col-0 wild-type plants were transformed with the cDNA of *HvD27* under the control of the endogenous *AtD27* promoter. For each transformation, three independent transgenic lines from the T3 generation that expressed the transgene were obtained (Supplementary Fig. S4a, b) and shoot branching was recorded at flowering. The *Atd27* mutants produced eight branches compared to three or four observed for Col-0 plants (Supplementary Fig. S4a, c). This enhanced branching in the *Atd27* mutant was reversed almost to the level of the untransformed controls in transgenic lines expressing the identified barley *HvD27* gene. In *pAtD27::HvD27*-transformed lines, *HvD27* expression resulted in an almost complete suppression of primary rosette-leaf branching in the Col-0 background (Supplementary Fig. S4a, c). ABA concentrations were analysed in leaves of 6-week-old Arabidopsis plants, and higher levels were found in the selected line *pAtD27::HvD27* B16-5 than in Col-0 (Supplementary Fig. S5).

For functional verification of the putative *HvMAX1* gene, the *Atmax1* mutant (Booker *et al.*, 2004) and Col-0 wild-type plants were transformed with the cDNA of *HvMAX1* under the control of the CaMV35S promoter. As the *HvMAX1* promotor sequence was yet not identified and AtMAX1 showed a relative strong and ubiquitous expression (Winter *et al.*, 2007), the CaMV35S promotor was chosen for the complementation approach. Three independent homozygous lines were recovered from the T3 generation of each transformation event and verified for transgene expression (Supplementary Fig. S6a, b). Enhanced branching of the *Atmax1* mutant was restored to Col-0 levels in the *35S::HvMAX1*/*Atmax1* transgenic lines, while in the Col-0 background *HvMAX1* expression led to an almost complete suppression of primary rosette-leaf branching (Supplementary Fig. S6a, c). These results demonstrate that *HvD27* and *HvMAX1* are able to complement the shoot branching phenotype of the corresponding Arabidopsis mutants and are able to restore the function of *AtD27* and *AtMAX1*, respectively.

### Tissue-dependent expression of strigolactone biosynthesis genes

To determine tissue-dependent expression of the four identified SL biosynthesis genes, qRT-PCR was performed on RNA isolated from roots, leaves, stem bases, and axillary buds of barley plants grown in a growth chamber for 10 weeks. All four genes were substantially expressed in roots (Supplementary Fig. S7), and *HvD27*, *HvCCD7,* and *HvCCD8* showed similar expression levels in the stem base. In contrast, *HvD27* and *HvMAX1* exhibited very high transcript levels in leaves, whereas *HvCCD7* and *HvCCD8* expression was found to be lower. In axillary buds, the mRNA levels of all four genes were low. These data are in agreement with a functional SL biosynthesis pathway in roots, where exceptionally high transcript levels of *HvMAX1* may point to a non-limiting role of this gene.

### Silencing *HvABA 8’-hydroxylase* genes induces a high-tillering phenotype

**Fig. 4.**
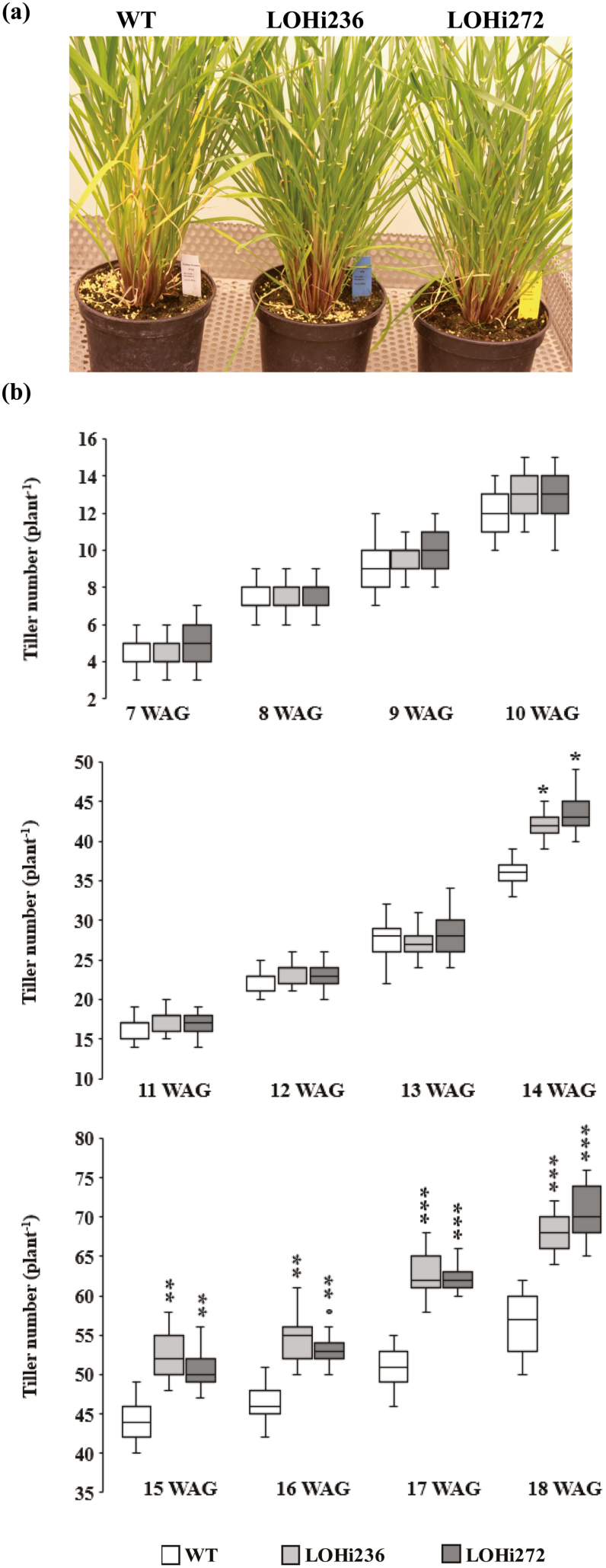
Tiller number for RNAi lines grown in the growth chamber. (a) The tillering phenotype in the wild-type (WT, Golden Promise) and the *HvABA 8’-hydroxylase* RNAi lines (LOHi236, LOHi272) at 16 weeks after germination (WAG). (b) Total tiller number per plant in the LOHi236 and LOHi272 transgenic lines compared with the wild-type from 7 to 18 WAG. of the box-plots represent the results from 20 plants per genotype. Significant differences as determined by Student’s *t*-test are indicated: *P˂0.05, **P˂0.01, ***P˂0.001.

Given the importance of SLs in inhibiting shoot branching and the interplay of ABA and SLs under abiotic stress conditions (López-Ráez *et al.*, 2010; Ha *et al.*, 2014), we examined shoot architecture in previously characterized transgenic barley plants expressing an RNAi construct for *HvABA 8’-hydroxylase* LOHi236 and LOHi272 (Seiler *et al.*, 2014). In these lines transgene expression is driven by the barley *late embryogenesis abundant* (*Lea*) *B19.3* gene promoter, which is active during the vegetative growth phase in root and shoot tissues (Supplementary Fig. S8). In previous work (Seiler *et al.*, 2014) it was shown that these plants have reduced expression of *HvABA 8’-hydroxylase* genes, which is responsible for ABA degradation, leading to altered levels of endogenous ABA. When cultured under controlled conditions in a growth chamber, shoot development of the transgenic lines was similar to the wild-type for the first 13 weeks of growth; however, from the 14th week onwards both transgenic lines produced significantly more tillers (Fig. 4). Due to continuous watering and nutrient supply, tillering continued until heading, when the LOHi236 and LOHi272 lines had produced approximately 10–15 tillers more than the wild-type.

To investigate whether this increase in final tiller number might result from differential regulation of axillary bud outgrowth or increased numbers of axillary meristems, the axillary meristems were examined at an early developmental stage. Sections of axillary meristem were taken at around 3–4 weeks after germination (WAG) and inspected microscopically. No differences were found in the anatomy or number between the LOHi lines and the Golden promise plants (Supplementary Fig. S9). When grown under greenhouse conditions, the LOHi lines showed a similar difference with more tillers, although the average number of developed tillers was reduced in all genotypes (Supplementary Fig. S10). The differences between greenhouse and growth chamber might have been caused by the different light conditions (Supplementary Table S4). In both cases, the difference in tiller formation became apparent at a relatively late developmental stage (12–14 WAG). Thus, enhanced tiller formation in *HvABA8’-hydroxylase*-suppressed lines was robust and reminiscent of a SL-deficient phenotype. Furthermore, increased tiller formation resulted in a lower number of spikes per plant, lower thousand-grain weight, and hence lower yield of the LOHi plants (Supplementary Fig. S11).

### ABA accumulation in the stem base and roots of LOHi lines

**Fig. 5.**
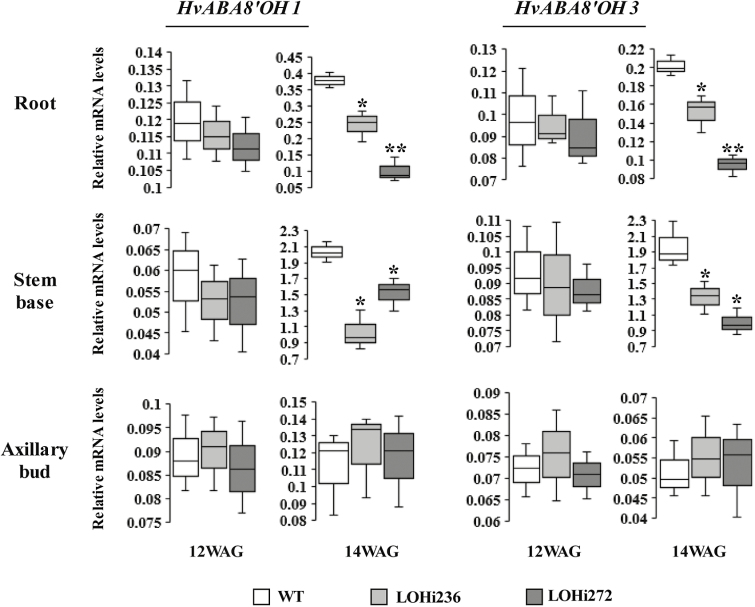
Relative transcript levels of the genes *HvABA8’OH1* and *HvABA8’OH3* in the stem base, roots, and axillary buds in wild-type (WT) plants and transgenic lines (LOHi236, LOHi272) at 12 and 14 weeks after germination (WAG). The box-plots represent the results from three independent biological samples. *Serine/threonine protein phosphatase PP2A-4* was used as the reference gene. Significant differences as determined by Student’s *t*-test are indicated: *P˂0.05, **P˂0.01.

To investigate the relationship between ABA homeostasis and the late-tillering phenotype, mRNA levels of ABA-related genes and ABA concentrations were determined in LOHi236 and LOHi272 and compared to untransformed wild-type Golden Promise controls. As quantified by RT-qPCR, the mRNA levels of *HvABA8’OH2* were relatively low in all tissues analysed, indicating that the encoded gene product might not significantly contribute to ABA degradation (Supplementary Fig. S12). The mRNA levels of the RNAi-targeted *HvABA8’OH1* and *HvABA8’OH3* genes were quantified in roots, stem bases, and axillary buds at 12 and 14 WAG (Fig. 5). At 12 WAG, when no significant difference in tiller formation was detectable, there was also no significant influence detectable of the RNAi construct on the relative mRNA levels of *HvABA8’OH1* and *HvABA8’OH3* in roots, stem bases, or axillary buds of LOHi plants. At 14 WAG, a clear increase of *HvABA8’OH1* and *3* transcript levels was detectable, which was significantly lower in roots and stem bases of LOHi plants compared to controls.

**Fig. 6.**
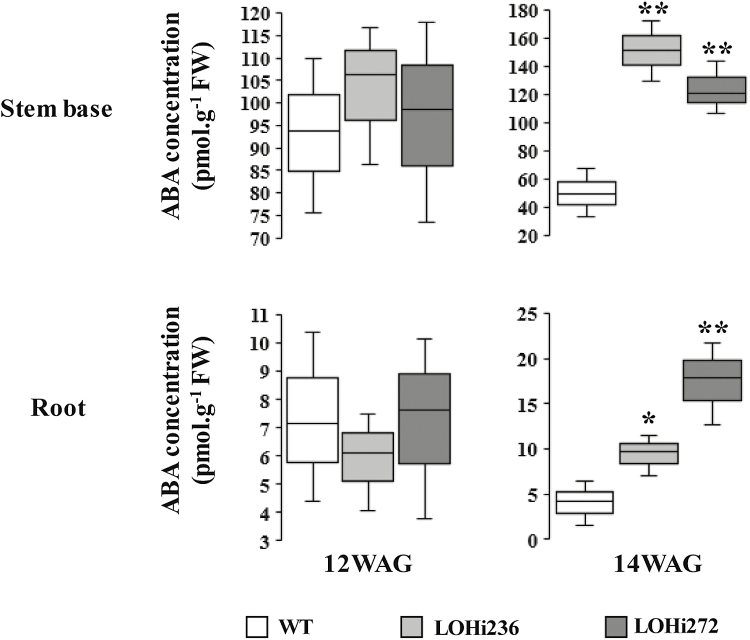
ABA concentrations in the stem base and roots of wild-type (WT) plants and LOHi antisense lines at 12 and 14 weeks after germination (WAG). The box-plots represent of the results from three independent biological replicates. Significant differences as determined by Student’s *t*-test are indicated: *P˂0.05, **P˂0.01.

LC-MS/MS analysis was used to determine ABA concentrations in the stem base and roots, In wild-type plants, the highest ABA concentration was detectable at 12 WAG in the stem base and no differences with the LOHi lines were detectable at this time. At 14 WAG the ABA concentration in the stem bases decreased in the wild-type (from 94 to 50 pmol g^–1^ FW), whereas mean concentrations increased in the LOHi lines (Fig. 6). This decrease was absent in the LOHi lines. In agreement with the reduced mRNA levels of *HvABA8’OH1* and *3*, ABA levels were ~70 % higher in the LOHi lines compared to the wild-type at 14 WAG. In roots, the detectable ABA content was ~10 times lower compared to the stem base. In line with higher ABA levels in the LOHi stem base at 14 WAG, higher ABA levels were also observed in root tissues.

### 5-deoxystrigol is lower in root exudates of LOHi lines

Although SLs have been detected in a variety of plant species, none have been identified in barley so far. To explore the identity of SLs in barley, LC-MS/MS analysis was performed. Barley plants were pre-cultured hydroponically, transferred to pots with sand, and subjected to phosphate starvation. Root exudates were collected, analysed, and the chromatograms were compared with retention times (RT) and mass transitions of available SL standards. As an example, a standard of 5-deoxystrigol was detected at RT 7.95 min in the MRM channel *m/z* 331.2>234.15, the internal standard D6-5-deoxystrigol was detected at RT 7.92 min in the MRM channel *m/z* 337.16>97.017, and 2’-*epi*-5-deoxystrigol was detected at RT 7.88 in the MRM channel *m/z* 331.2>234.0 (Supplementary Fig. S13). The chromatogram of a crude root exudate revealed an intense peak in the channel *m/z* 331.2>216.15 at RT 7.95 min, which matched the standard of 5-deoxystrigol.

**Fig. 7.**
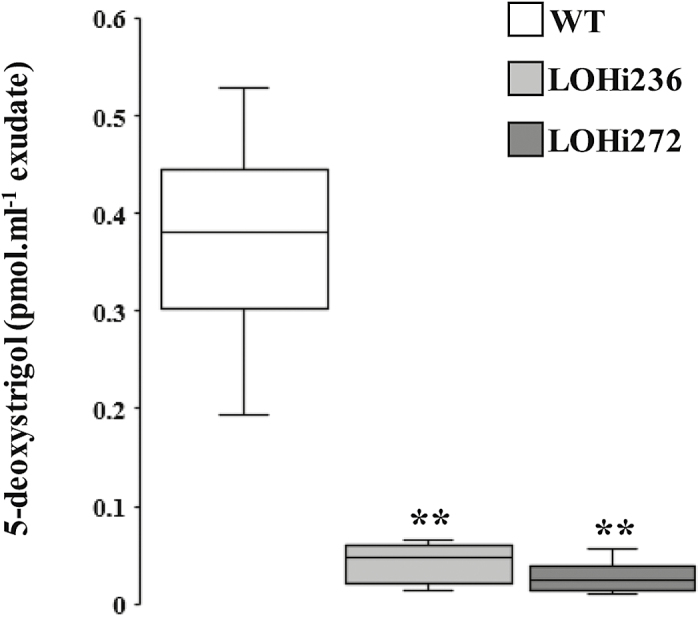
Strigolactone concentrations in barley root exudates as determined by MRM-LC-MS/MS analysis. The data show the concentrations of 5-deoxystrigol in root exudates of LOHi (LOHi236 and LOHi272) and wild-type plants (WT) after 7 d of phosphate starvation, which commenced at 14 weeks after germination. The box-plots represent of the results from five independent biological replicates. Significant differences as determined by Student’s *t*-test are indicated: **P˂0.01.

To investigate the abundance of detectable SLs in the LOHi lines, plants were pre-cultured hydroponically, transferred to pots with sand and subjected to phosphate starvation and root exudate analysis. 5-deoxystrigol was significantly (*P*<0.01) lower in root exudates of LOHi236 and LOHi272 compared with the wild-type at 14 WAG (Fig. 7). This lower abundance coincided with increased tiller formation in the LOHi lines.

**Fig. 8.**
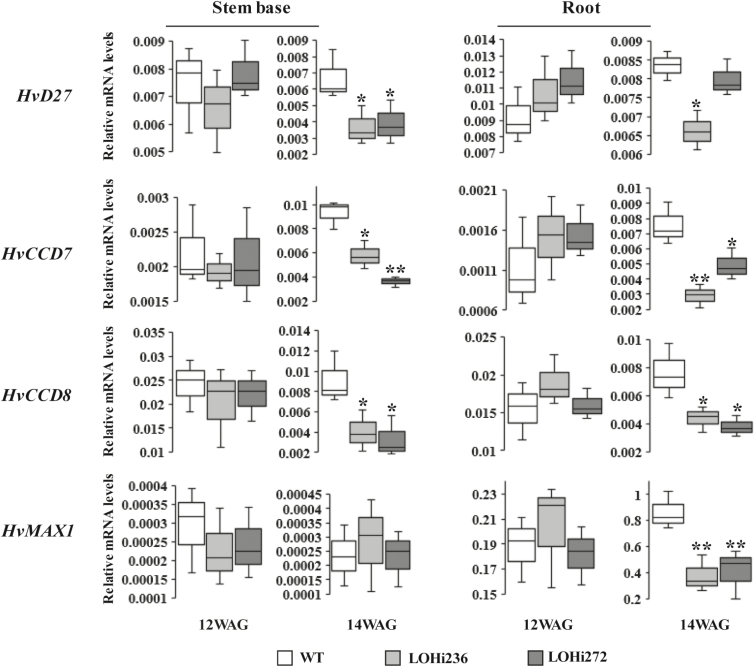
Transcript levels of genes from the SL biosynthetic pathway in stem bases and roots of wild-type (WT) plants and LOHi antisense lines at 12 and 14 weeks after germination (WAG). The box-plots represent of the results from three biologically independent samples. *Serine/threonine protein phosphatase PP2A-4* was used as the reference gene. Significant differences as determined by Student’s *t*-test are indicated: *P˂0.05, **P˂0.01.

To corroborate a functional link between ABA and *HvD27*, *HvMAX1*, *HvCCD7*, and *HvCCD8*, gene expression was analysed using qRT-PCR. At 12 WAG, no differences between the LOHi lines and wild-type plants were observed for the mRNA abundance of all four genes in the stem base and root tissue (Fig. 8). At 14 WAG, the relative mRNA levels of *HvD27*, *HvCCD7*, and *HvCCD8* were found to be significantly lower in the stem base and root tissue of the LOHi lines compared to the wild-type, although there was inconsistency in the LOHi lines in the root tissue. *HvMAX1*, which in contrast to the other genes was only clearly detectable in roots at later time points, showed a tissue-specific reduction of transcript levels in LOHi plants.

## Discussion

ABA and SLs are plant hormones involved in the regulation of growth. Both share a common precursor (all-*trans*-β-carotene) but knowledge of the genes involved in SL biosynthesis in barley is limited. Here, we identified four genes involved in SL biosynthesis in barley and identified 5-deoxystrigol in root exudates. Based on analysis of transgenic plants with suppressed ABA degradation, we have shown that there is cross-talk between ABA and SL biosynthesis that controls tillering in barley. Thus, the present study provides for the first time genetic evidence for an antagonistic interaction between ABA and SLs in plants.

### Identification of *HvD27*, *HvCCD7*, *HvCCD8*, and *HvMAX1* and their involvement in SL biosynthesis

Despite the importance of SLs for shoot architecture in plants, little information is available for this class of plant hormones in cereal species other than rice. Based on amino acid similarities to homologous proteins in rice and Arabidopsis, *HvD27*, *HvCCD7*, *HvCCD8*, and *HvMAX1* were identified in barley and confirmed by phylogenetic analysis to represent the closest homologs of their functionally characterized counterparts in these two species (Supplementary Fig. S2). Based on their tissue-specific mRNA abundance, *HvD27*, *HvCCD7*, and *HvCCD8* were highly expressed in both the stem base and roots at the time points examined (Fig. 8). However, the newly identified gene *HvMAX1* was only expressed in roots, suggesting a role in the root-specific conversion of carlactone to carlactonic acid and further to 5-deoxystrigol, which was subsequently detected in barley root exudates (Fig. 7). Such a pathway is in agreement with the reported ability of barley root exudates to stimulate germination of *Striga* (Steinkellner *et al.*, 2007) and reminiscent of SL species found in Arabidopsis or *Nicotiana benthamiana* (Marzec and Muszynska, 2015). By contrast, orobanchol-type SLs have been identified as the predominant forms in rice (Xie *et al.*, 2010).

To verify the proposed gene function in SL biosynthesis, virus-induced gene silencing was performed to silence *HvCCD7* (Fig. 2) and *HvCCD8* (Fig. 3) *in planta*. This post-transcriptional gene silencing resulted in increased tiller formation, which is in agreement with the proposed involvement of *HvCCD7* and *HvCCD8* in SL production. For functional analysis of *HvD27* and *HvMAX1,* heterologous complementation was performed employing the corresponding *Arabidopsis thaliana* mutant lines (Booker *et al.*, 2004; Waters *et al.*, 2012). Functional complementation was achieved by ectopic expression of *HvD27* and *HvMAX1*. Both barley genes were able to complement the highly branched phenotype of the respective Arabidopsis mutants back to wild-type levels (Supplementary Figs S4, S6). Due to the lack of sequence information this approach could not be applied to *HvCCD7* and *HvCCD8*.

To further validate the function of the identified genes in tiller formation, transgenic LOHi plants with suppressed hydroxylation of ABA were examined. These plants showed a tissue-specific and development-specific suppression of *HvD27*, *HvCCD7*, *HvCCD8*, and *HvMAX1* that temporally coincided with enhanced tiller formation (14 WAG, Fig. 4). This reduction in transcript levels of SL biosynthesis genes was accompanied by lower abundance of 5-deoxystrigol in root exudates of P-starved barley plants (Fig. 7). Taken together, these results support the physiological function of the identified genes in SL biosynthesis. According to previous results in Arabidopsis (Brewer *et al.*, 2016), the genes identified here are likely to represent core components of the SL biosynthesis pathway in barley.

In addition to the SL biosynthesis genes discussed here, further genes are involved in SL signaling such as *D14*, *MAX2*, and *D53* (Al-Babili and Bouwmeester, 2015). As pointed out by Brewer *et al.* (2016), knowledge of bioactive SLs is limited; therefore, identification of additional genes is required. Recently, *HvD14* has been identified and characterized as a component involved in SL signaling in barley. A mutation in this gene causes a larger number of tillers than in wild-type plants (Marzec *et al.*, 2016).

### Involvement of 5-deoxystrigol in tiller formation in barley

5-deoxystrigol has been proposed as a common precursor of various SLs (Rani *et al.*, 2008; Zwanenburg *et al.*, 2009). It is widely distributed in the plant kingdom and has been detected in root exudates from both monocots (Awad *et al.*, 2006) and dicots (Yoneyama *et al.*, 2007). Using LC-MS/MS, we exclusively identified 5-deoxystrigol in root exudates of barley. Other known SLs could not be detected or were below the detection limits. Root exudates of barley have been reported to stimulate germination of *Striga* and *Orobanche* seeds (Steinkellner *et al.*, 2007). However, we cannot rule out the possibility that other unknown SLs, including orobanchol-type, could be synthesized and released via root exudates in barley.

As SLs are of low abundance and difficult to detect even with sensitive LC-MS/MS devices, plants were subjected to P-starvation prior to collection of root exudate, since P-deficiency promotes SL biosynthesis and release (Al-Babili and Bouwmeester, 2015). In all studies reported so far, the amount of detectable SLs in root exudates (also sampled after P-starvation) has correlated with the inhibition of above-ground lateral organ outgrowth (Brewer *et al.*, 2013; Borghi *et al.*, 2016). We took the detectable amount of 5-deoxystrigol in root exudates as proxy for 5-deoxystrigol biosynthesis in the roots. Supported by the root-specific expression of *HvMAX1*, we propose that the formation of 5-deoxystrigol is confined to roots, as reflected by its occurrence in the root exudate. Whether 5-deoxystrigol or other bioactive SLs might be translocated to the stem base cannot be concluded based on the present results. To date, 5-deoxystrigol is the first member of this class of plant hormones identified in barley.

### Interaction between abscisic acid and strigolactones occurs at the level of SL biosynthesis

**Fig. 9.**
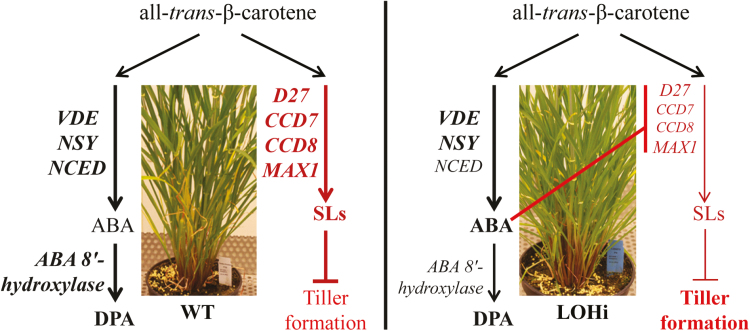
Schematic model of the regulatory interaction between ABA and SL in tiller formation. The wild-type (WT) plants maintain hormonal homeostasis between ABA and SL. In contrast, in the LOHi lines transiently high endogenous ABA levels triggered by a spatial-temporal silencing of the *HvABA 8’-hydroxylase* genes represses SL biosynthesis, mainly via down-regulation of *HvCCD7/8*, which results in low SL production. This subsequently releases the inhibition of SL, and finally causes a later, high-tillering phenotype. Abbreviations: VDE, violaxanthin de-epoxidase; NSY, neoxanthin synthase; NCED, 9-*cis*-epoxycarotenoid dioxygenase; D27, dwarf 27; CCD7/8, carotenoid cleavage dioxygenase 7/8; MAX1, more axillary growth 1; DPA, dihydrophaseic acid.

In the present work, we choose a genetic approach to investigate ABA–SL interactions. We employed the transgenic barley lines LOHi236 and LOHi272, in which transcript levels of *HvABA 8’-hydroxylase* genes are post-transcriptionally suppressed by an RNAi construct (Seiler *et al.*, 2014). In these lines, transcript levels of *HvABA 8’-hydroxylase1* and *3* were strongly decreased in the stem base tissue at 14 WAG, confirming that silencing resulted in down-regulation of *HvABA 8’-hydroxylase1* and *3* (Fig. 5). In the stem base, effective post-transcriptional gene silencing was most likely responsible for enhanced tiller formation, because suppression of these genes caused reduced degradation of ABA and resulted in three-fold higher ABA levels at 14 WAG (Fig. 6). In line with this, phenotypic differences were detected 14 WAG, but not earlier. This was probably due to (1) the *Lea* gene promoter that we used driving expression of the RNAi silencing inducer at this late developmental phase (Supplementary Fig. S8), and (2) the development-specific expression pattern of the target genes (Fig. 5). In several studies a negative association has been reported between ABA and axillary bud development. In Arabidopsis buds, increased expression of ABA-associated genes under light with a low R:FR ratio results in suppressed stem branching (González-Grandío *et al.*, 2013). Therefore, we initially expected that elevated ABA levels would suppress tiller formation. However, elevated ABA accumulation in the roots and stem base of both LOHi lines clearly coincided with higher tiller numbers (Figs 4, 6, Supplementary Fig. S10). ABA is therefore unlikely to be directly involved in tiller formation. Instead, we conclude that lower SL biosynthesis, as a result of increased ABA, is responsible for the observed phenotype, since SLs have been characterized as suppressors of lateral shoot branching (Borghi *et al.*, 2016). The possibility that SL biosynthesis is regulated by ABA has already been suggested by exogenous application of ABA to Arabidopsis plants, leading to a decrease in the transcript levels of the SL biosynthesis genes *CCD7* and *CCD8* (Ha *et al.*, 2014). In the present transgenic approach, the major change in ABA levels was detected in the stem base, but also in roots, although to a lesser extent (Fig. 6). This coincided with a significant reduction of SL biosynthesis-related transcript levels (Fig. 8). Thus, we conclude that the elevated ABA levels in these tissues caused the transcriptional down-regulation of genes involved in SL biosynthesis. Interestingly, substantial expression of *HvMAX1* was only detectable in wild-type roots. However, at 14 WAG transcript levels had increased in the wild-type but not in the LOHi lines. These genotype-, tissue-, and developmental-specific differences are in agreement with the detected differences in 5-deoxystrigol production (Fig. 7). Based on our findings here, we propose a model for the regulatory interaction between ABA and SLs in tiller formation (Fig. 9). In wild-type plants, homeostasis between ABA and SLs is important for reduced tiller formation after flowering. A transiently elevated endogenous level of ABA, as triggered by the silencing of the *HvABA 8’-hydroxylase* genes, represses SL biosynthesis mainly via down-regulating the transcript levels of *HvD27*, *HvCCD7*, *HvCCD8*, and *HvMAX1*, which results in less SL production. As a consequence, lower 5-deoxystrigol levels result in reduced tiller suppression, which causes the observed high-tillering phenotype of the LOHi lines at later developmental stages.

### *HvD27* as the target for ABA-dependent SL biosynthesis

Based on low SL levels of the corresponding mutant, OsD27 has been identified as an iron-containing enzyme involved in SL biosynthesis (Lin *et al.*, 2009). The purified enzyme exerts isomerase activity that is required for the conversion of all-*trans*-β-carotene to 9-*cis*-β-carotene in a reversible manner (Alder *et al.*, 2012). All-*trans*-β-carotene is the shared common precursor for ABA and SL production. The 9-*cis*/all-*trans* isomerase activity of D27 raised the question whether this enzyme might also be involved in ABA biosynthesis by pulling all-*trans*-β-carotene into ABA biosynthesis (Fig. 1). Recently, this activity was confirmed by a study on the substrate specificity of the rice SL biosynthesis enzyme DWARF27 in carotenoid-accumulating *E. coli* strains and in *in vitro* assays performed with the recombinant OsD27 enzyme (Bruno and Al-Babili, 2016) (Fig. 1). This finding is further supported by the observation that increasing HvD27 activity in Arabidopsis by ectopic overexpression resulted in an increase of ABA, modulating ABA homoeostasis (Supplementary Fig. S5). This increase was far below the ABA levels typically associated with drought stress (~20–40 ng ABA g^–1^ FW; Cho *et al.*, 2008; Liu *et al.*, 2014) and it was not accompanied by any visual phenotype, and may therefore be subject to the complex autoregulation of ABA homoeostasis. Considering that SLs are mostly synthesized in roots with minor synthesis in shoots (Al-Babili and Bouwmeester, 2015) and that in barley *HvMAX1* is predominately expressed in roots (Fig. 8), we propose that the major site of the interaction between ABA and SL is the root tissue. To what extent ABA-mediated suppression of SL biosynthesis genes in the stem base contributed to enhanced tiller outgrowth requires further investigation.

The isomerase activity of HvD27 separates the SL pathway from ABA biosynthesis and is thus an ideal target for hormonal interaction. On the one hand, ABA may most effectively suppress the complete SL biosynthesis pathway by only regulating HvD27. On the other hand, suppression of *HvD27* may favor ABA biosynthesis via higher availability of the precursor and thus become part of the auto-regulatory pathway in ABA homeostasis.

Taken together, the interaction between ABA and SLs identified here may play an important role in agricultural plant production. Tiller formation at later developmental stages is an undesired trait, because late tillers rarely contribute to grain yield. SL production especially at later developmental stages may help to avoid surplus tiller formation, in particular when ABA levels are low. The ABA–SL interaction may represent an important mechanism that suppresses the generation of unproductive tillers.

## Supplementary data

Supplementary data are available at *JXB* online.

Fig. S1. Genomic organization of BSMV RNA γ modified to express antisense gene-specific fragments.

Fig. S2. Phylogenetic trees of amino acid sequences of proteins in the SL biosynthesis pathway and barley genomic map with relative physical positions of the genes investigated in this study.

Fig. S3. Virus-induced gene silencing of *HvCCD7* in barley.

Fig. S4. Genetic complementation of the Arabidopsis *d27* mutant with the *Hordeum vulgare D27* gene.

Fig. S5. ABA concentrations in 6-week-old Arabidopsis leaves of Col-0 and the transgenic line B16-5 expressing the *pAtD27::HvD27* construct in the Col-0 background.

Fig. S6. Genetic complementation of the Arabidopsis *max1* mutant with the *Hordeum vulgare MAX1* gene.

Fig. S7. Expression of genes from the SL biosynthesis pathway across various tissues and organs in barley wild-type plants, together with selected reference genes.

Fig. S8. Expression levels of *late embryogenesis-abundant* (*Lea*) *B19.3* in barley various tissues and across the life cycle of barley.

Fig. S9. Development of axillary meristems in wild-type and LOHi antisense lines.

Fig. S10. Variation in tiller numbers with time under greenhouse conditions.

Fig. S11. Spike number per plant and thousand-grain weight at maturity under greenhouse conditions.

Fig. S12. Expression levels of the genes *HvABA8’OH 1*, *2*, and *3* in the roots, stem base, leaves, and axillary buds in wild-type plants at 10 WAG.

Fig. S13. MRM-LC-MS/MS analysis of strigolactones identity in barley root exudates.

Fig. S14. Plasmid maps of pd35S-Nos-AB-M and p6U10.

Table S1. List of primer sequences for PCR, RT-PCR, and quantitative RT-PCR.

Table S2. Details of the Hoagland’s nutrient solution.

Table S3. List of protein sequences used in the phylogenetic analysis.

Table S4. Details of growth conditions used for the tiller phenotyping of the LOHi lines.

Supplementary Table S1

Supplementary Table S2

Supplementary Table S3

Supplementary Table S4

Supplementary Figures 1

Supplementary Figures 2a

Supplementary Figures 2b

Supplementary Figures 2c

Supplementary Figures 3

Supplementary Figures 4

Supplementary Figures 5

Supplementary Figures 6

Supplementary Figures 7

Supplementary Figures 8a

Supplementary Figures 8b

Supplementary Figures 9

Supplementary Figures 10

Supplementary Figures 11

Supplementary Figures 12

Supplementary Figures 13

Supplementary Figures 14
