# Corrigendum to: Abscisic acid influences tillering by modulation of strigolactones in barley

**DOI:** 10.1093/jxb/ery306

**Published:** 2018-08-28

**Authors:** Hongwen Wang, Wanxin Chen, Kai Eggert, Tatsiana Charnikhova, Harro Bouwmeester, Patrick Schweizer, Mohammad R Hajirezaei, Christiane Seiler, Nese Sreenivasulu, Nicolaus von Wirén, Markus Kuhlmann

**Affiliations:** 1 Department of Physiology and Cell Biology, Leibniz Institute of Plant Genetics and Crop Plant Research (IPK) Gatersleben, Corrensstrasse, Stadt Seeland, Germany; 2 Department of Breeding Research, Leibniz Institute of Plant Genetics and Crop Plant Research (IPK) Gatersleben, Corrensstrasse, Stadt Seeland, Germany; 3 Laboratory of Plant Physiology, Wageningen University, Droevendaalsesteeg, Wageningen, The Netherlands; 4 Plant Hormone Biology Group, Swammerdam Institute for Life Sciences, University of Amsterdam, XH Amsterdam, The Netherlands; 5 Department of Molecular Genetics, Leibniz Institute of Plant Genetics and Crop Plant Research (IPK) Gatersleben, Corrensstrasse, Stadt Seeland, Germany; 6 International Rice Research Institute (IRRI), Grain Quality and Nutrition Center, Metro Manila, Philippines

*Journal of Experimental Botany*, Vol. 69, No. 16 pp. 3883–3898, doi:10.1093/jxb/ery200

The authors of this article would like to give due credit to a colleague, Dr. Götz Hensel, whose assistance was of great value to this study. The online version of this article has now been updated to reflect that contribution.

The authors would like to apologise for the original omission.

