# Supplementary figures and images for: Abscisic acid influences tillering by modulation of strigolactones in barley
Source: J Exp Bot. 2018 Jul 5;69(16):3883–98. doi: 10.1093/jxb/ery200 (PMC6054196; doi:10.1093/jxb/ery200)

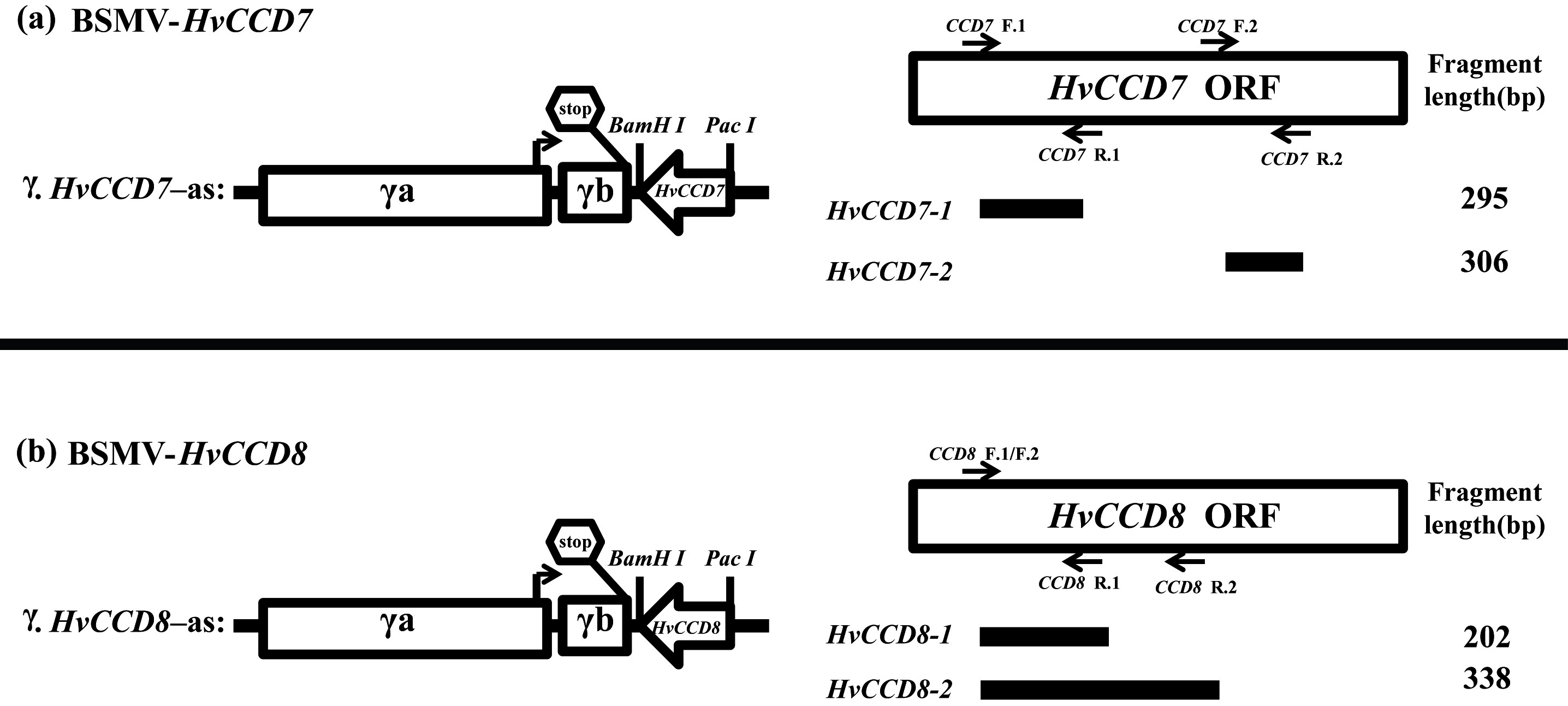

Supplement: Supplementary Figures 1 [file ery200_suppl_supplementary_figures_1.jpeg]

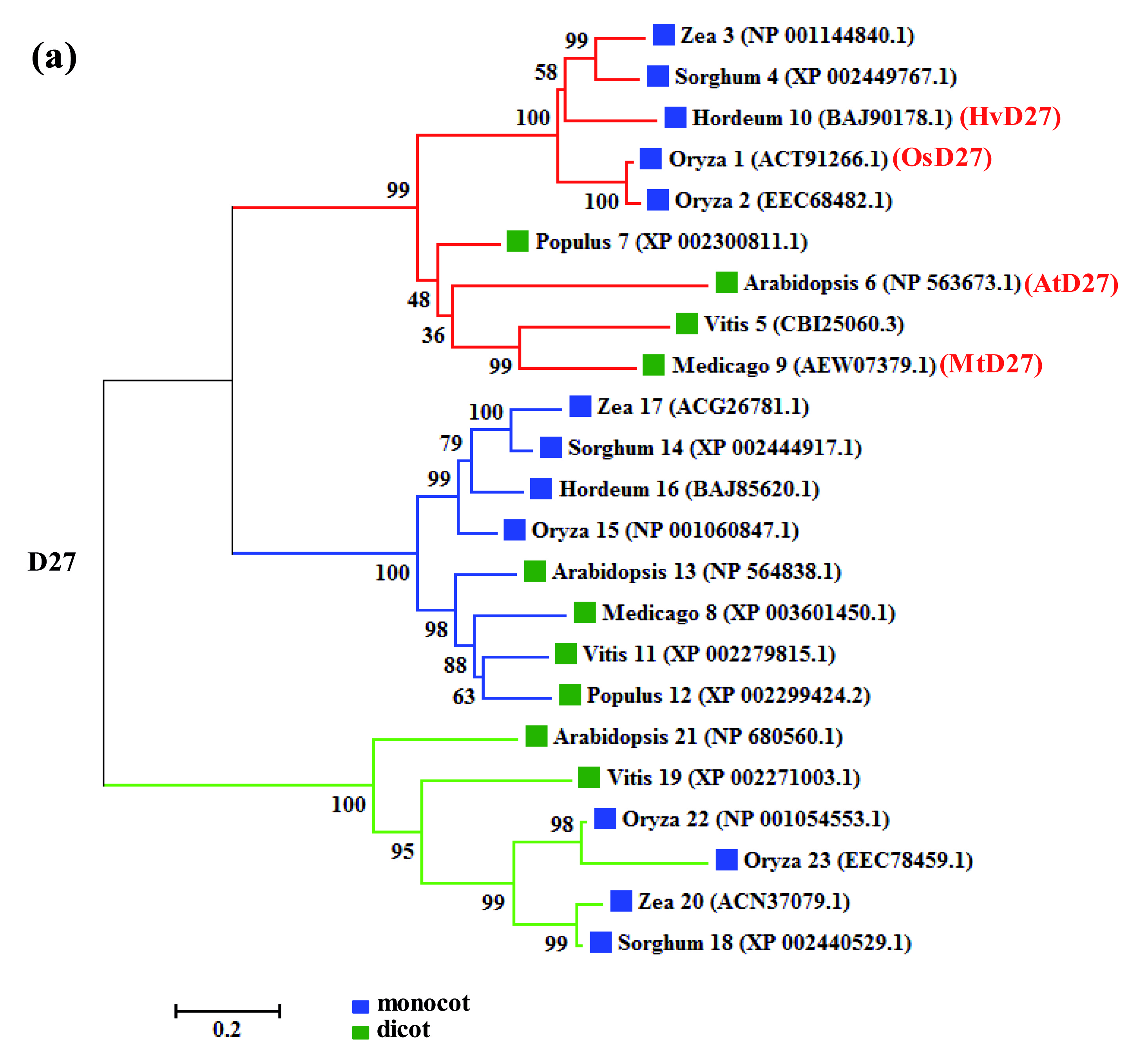

Supplement: Supplementary Figures 2a [file ery200_suppl_supplementary_figures_2a.jpeg]

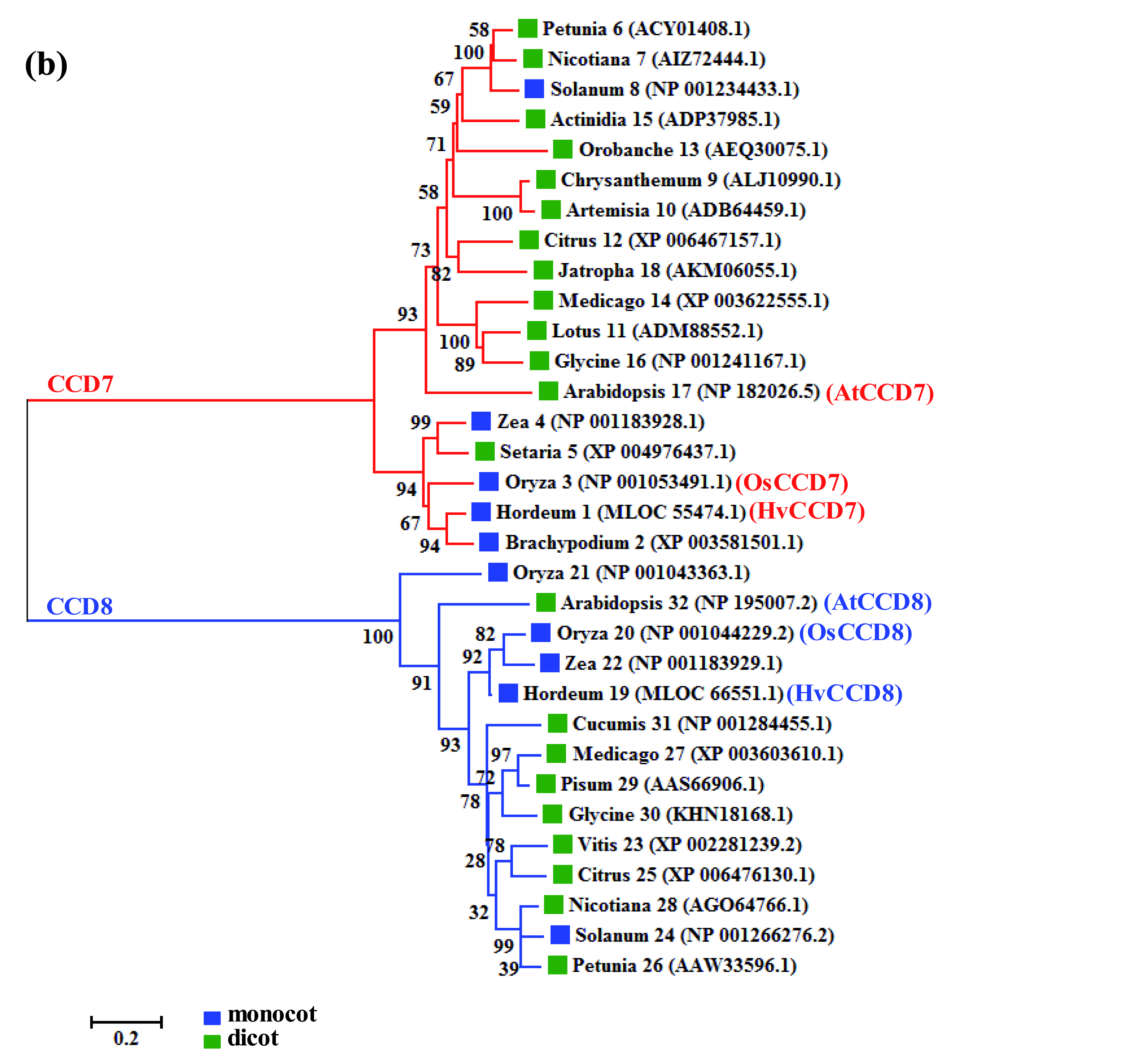

Supplement: Supplementary Figures 2b [file ery200_suppl_supplementary_figures_2b.jpeg]

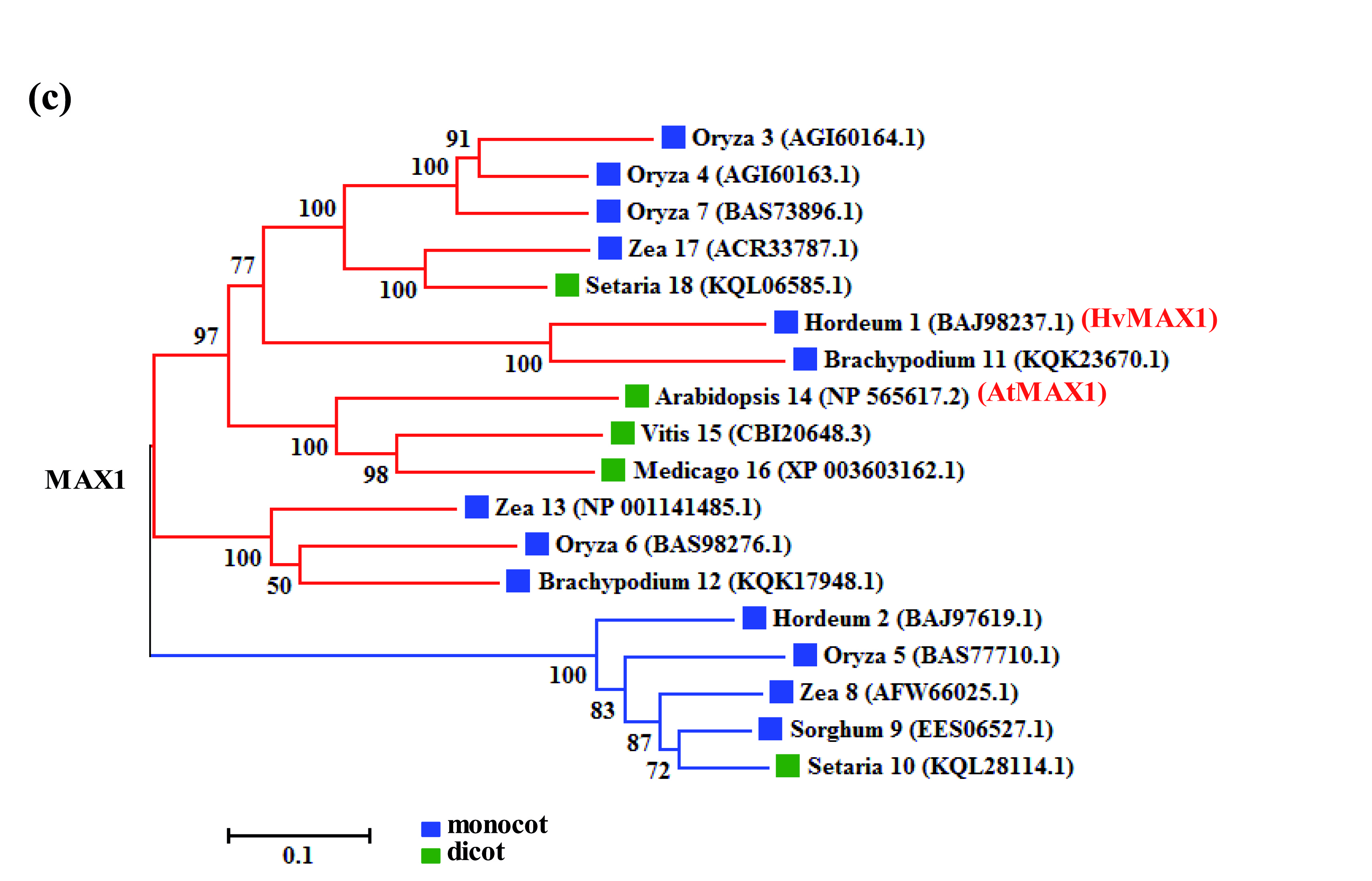

Supplement: Supplementary Figures 2c [file ery200_suppl_supplementary_figures_2c.jpeg]

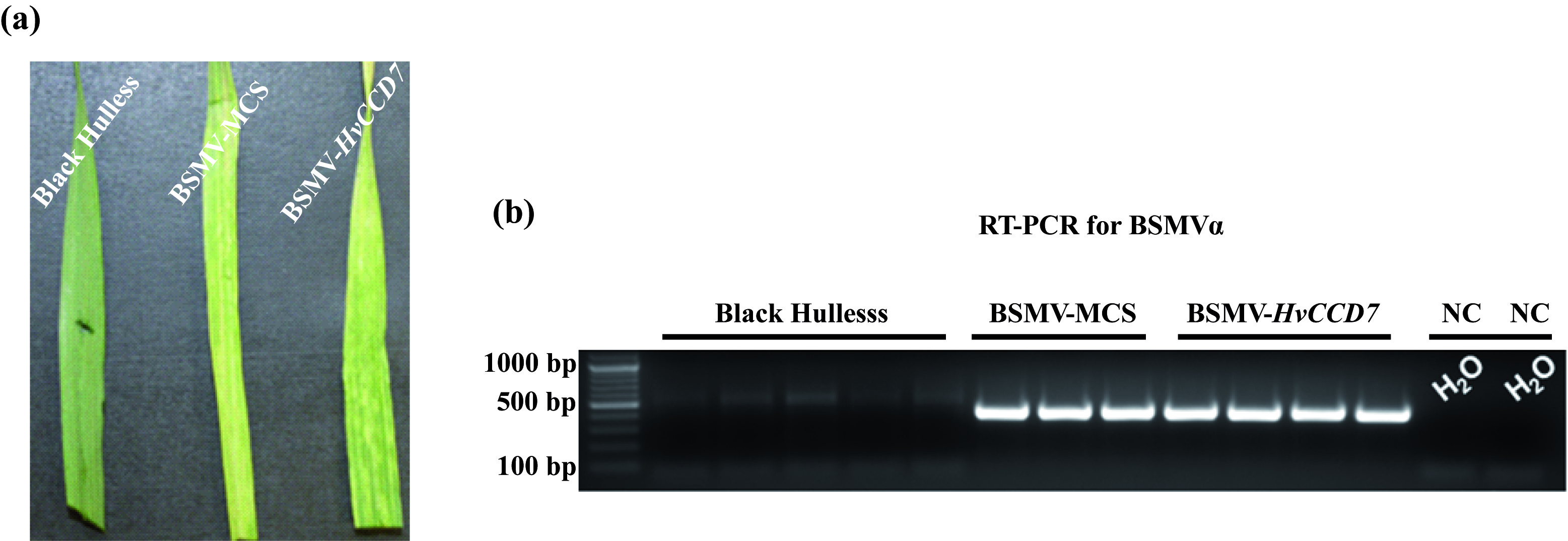

Supplement: Supplementary Figures 3 [file ery200_suppl_supplementary_figures_3.jpeg]

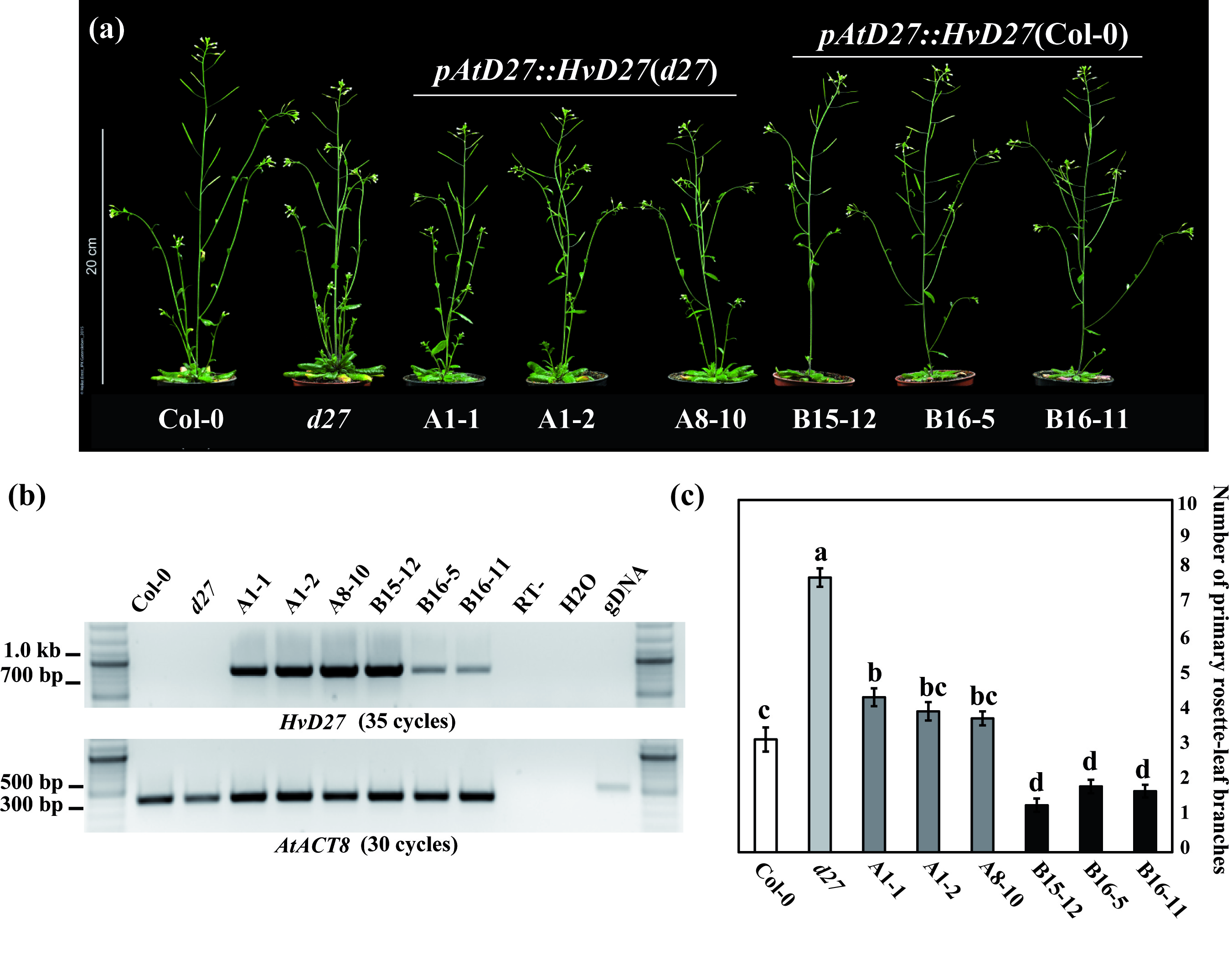

Supplement: Supplementary Figures 4 [file ery200_suppl_supplementary_figures_4.jpeg]

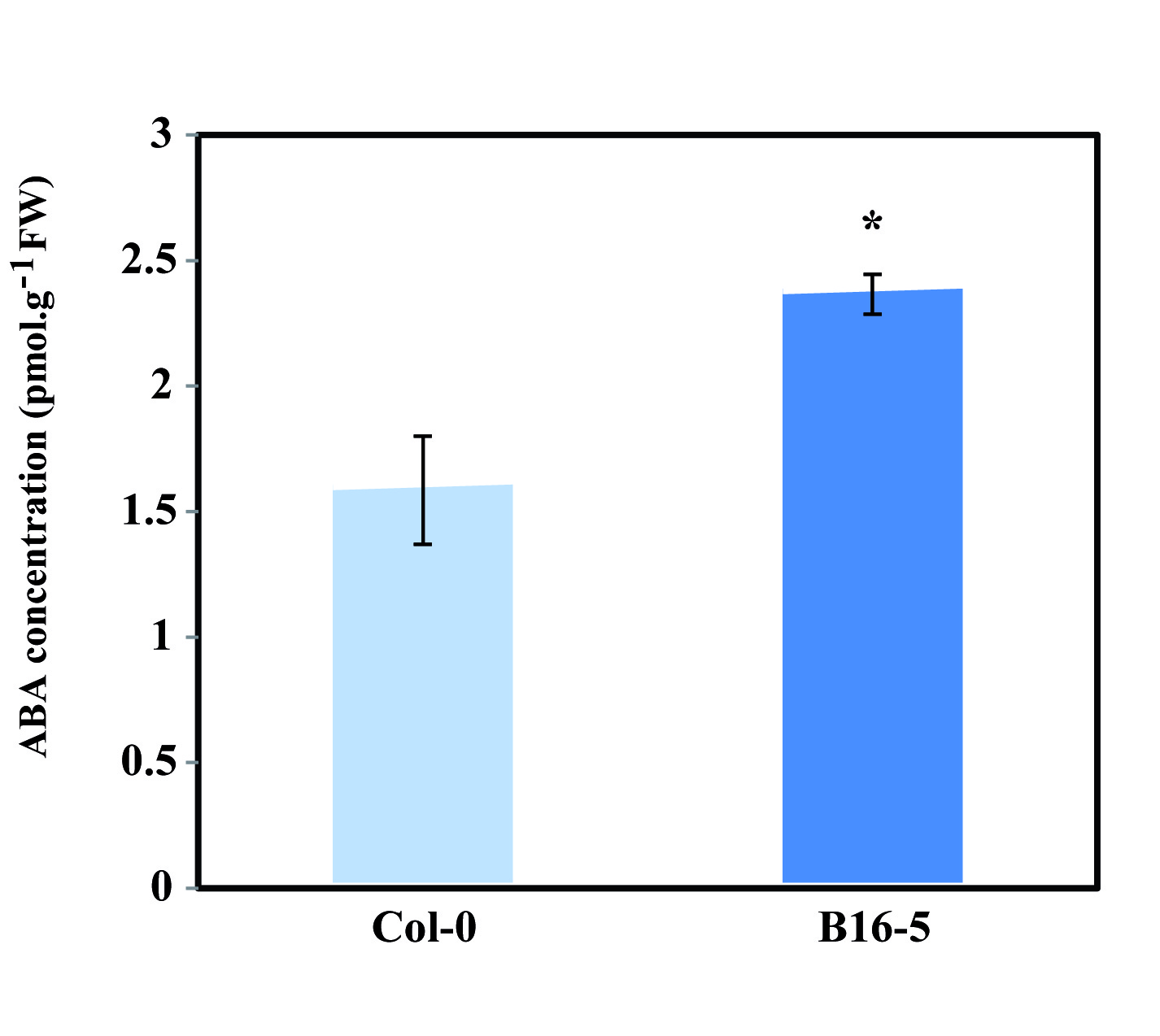

Supplement: Supplementary Figures 5 [file ery200_suppl_supplementary_figures_5.jpeg]

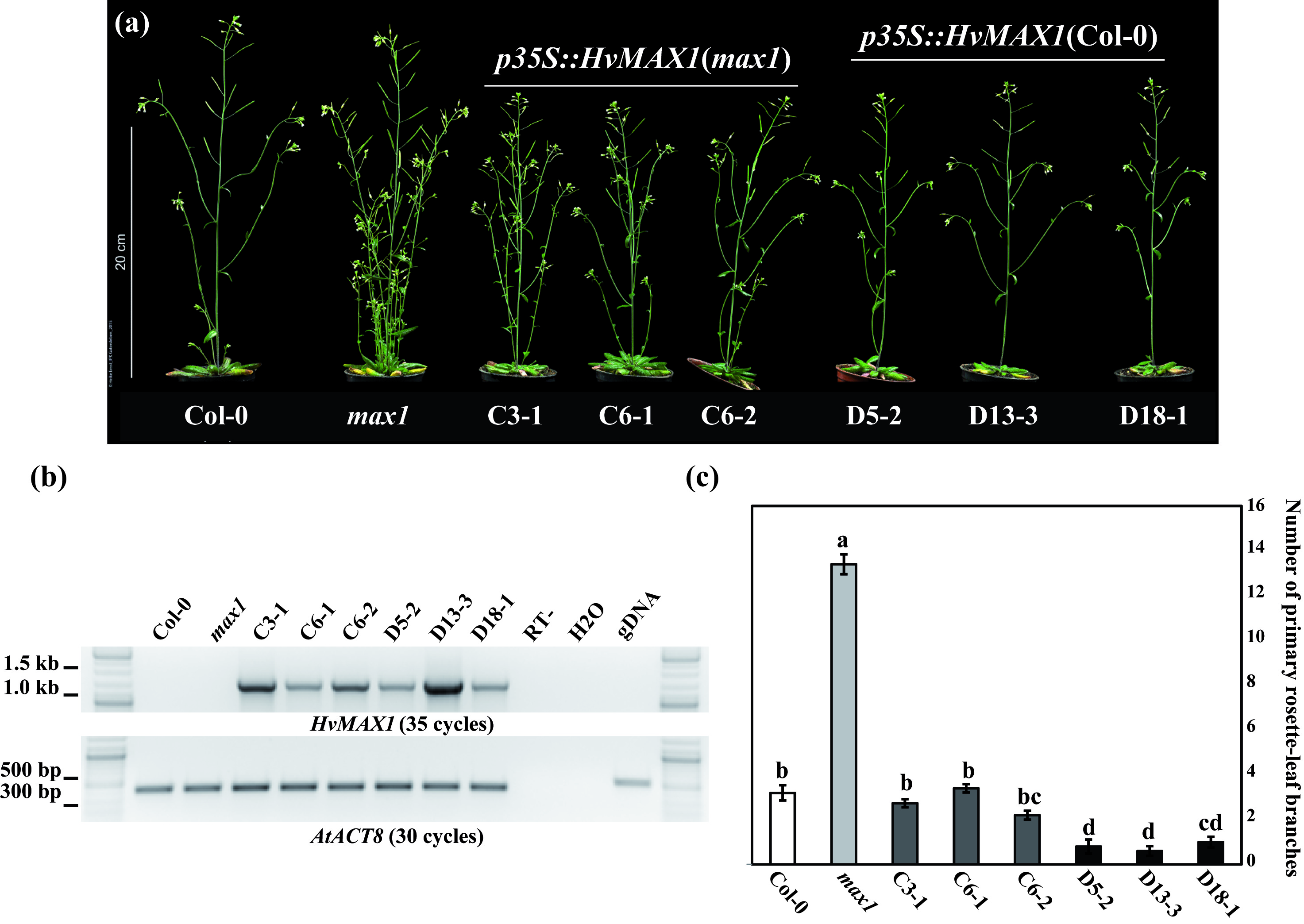

Supplement: Supplementary Figures 6 [file ery200_suppl_supplementary_figures_6.jpeg]

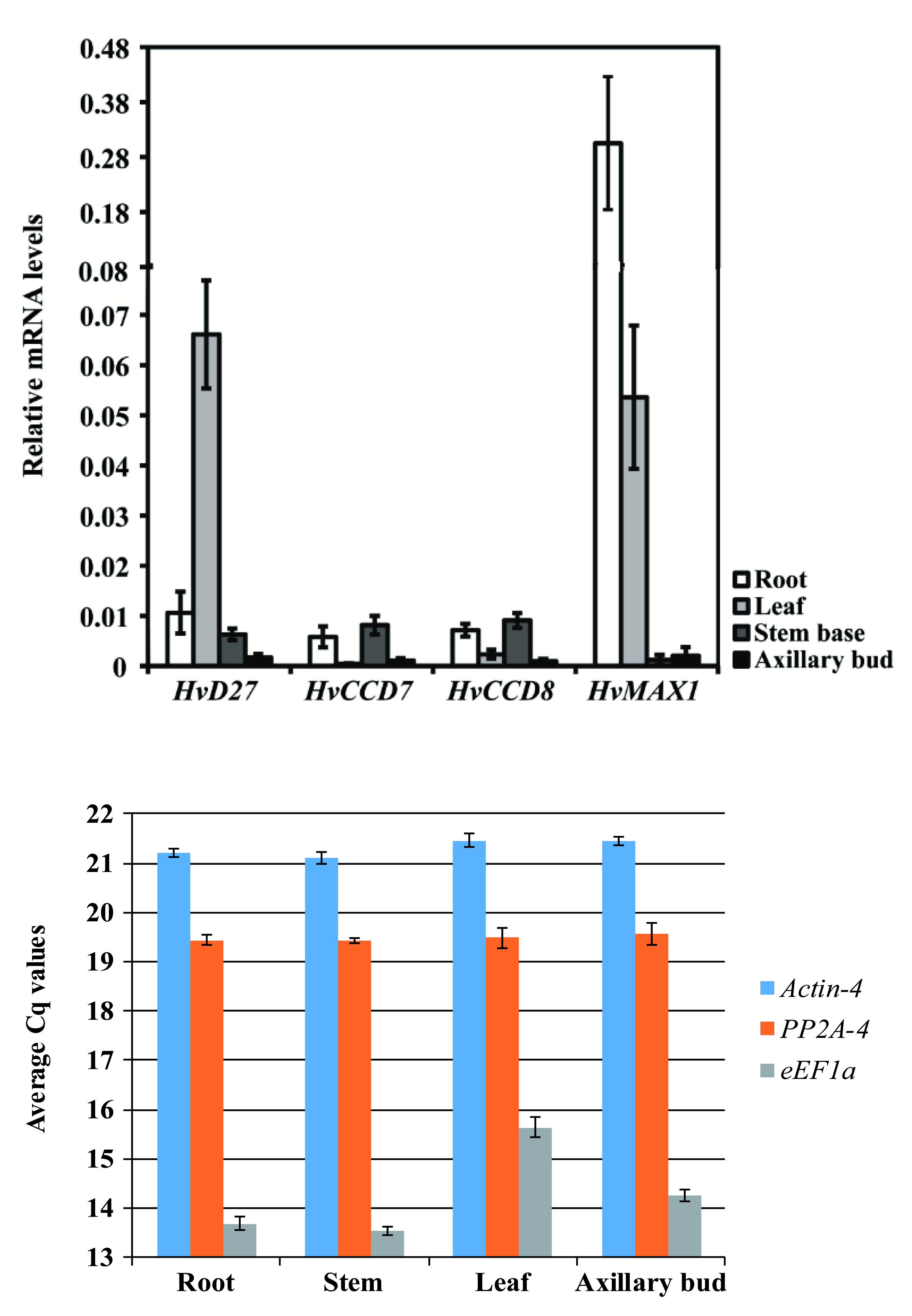

Supplement: Supplementary Figures 7 [file ery200_suppl_supplementary_figures_7.jpeg]

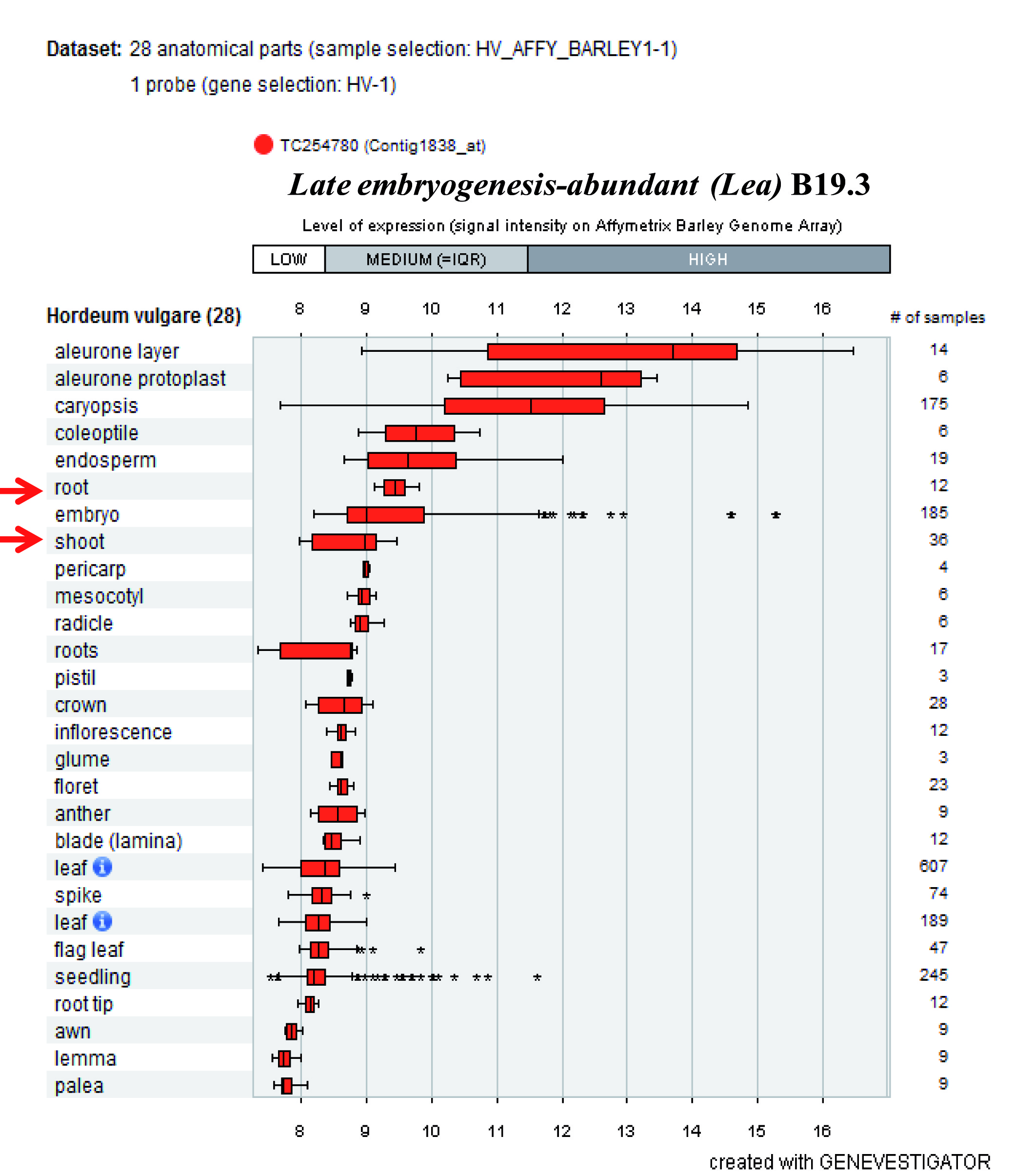

Supplement: Supplementary Figures 8a [file ery200_suppl_supplementary_figures_8a.jpeg]

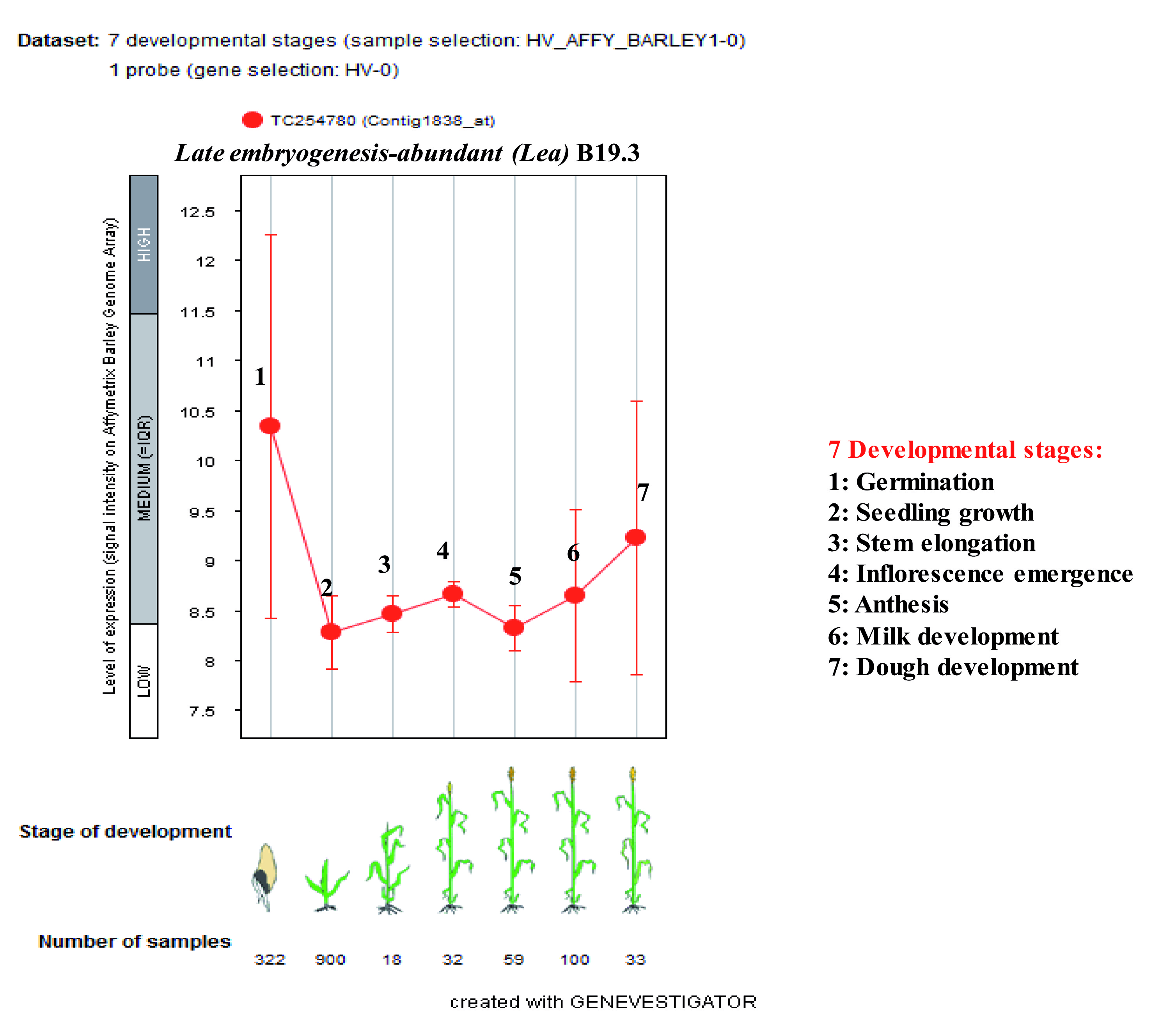

Supplement: Supplementary Figures 8b [file ery200_suppl_supplementary_figures_8b.jpeg]

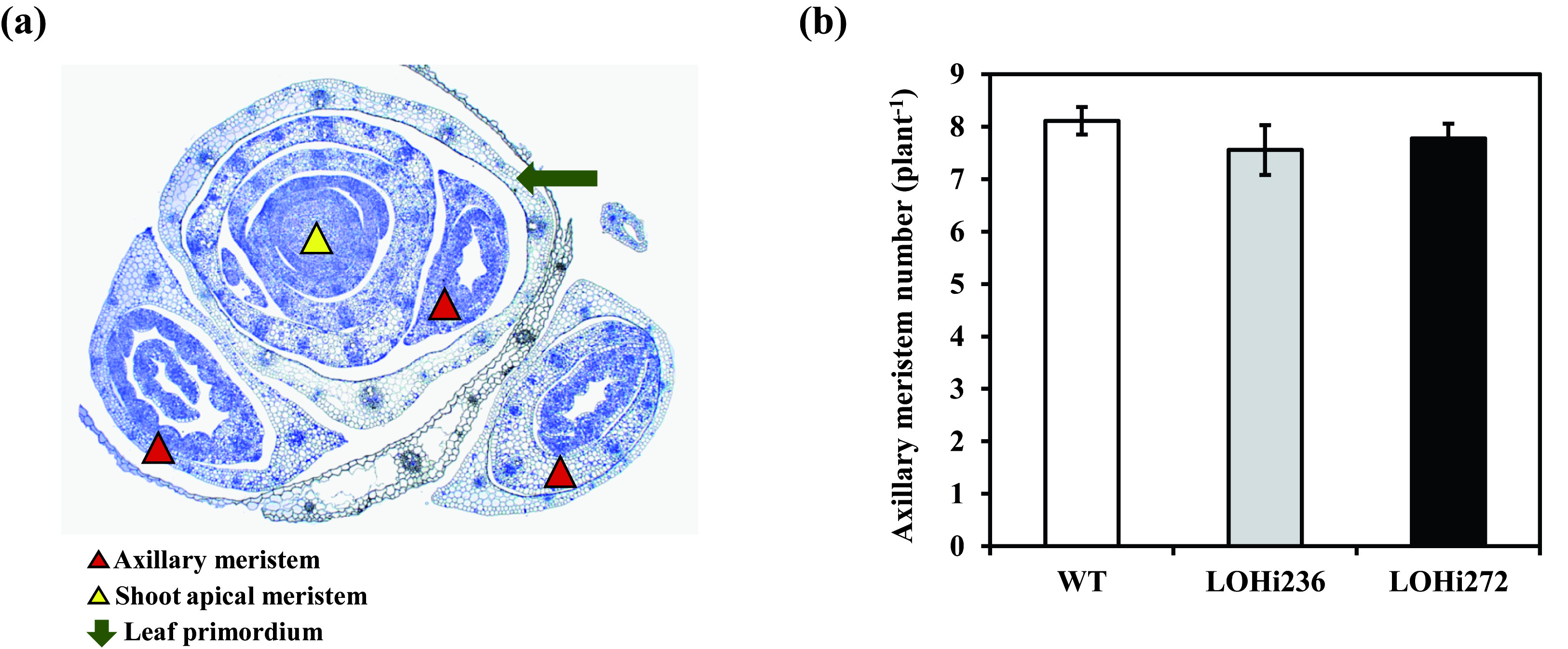

Supplement: Supplementary Figures 9 [file ery200_suppl_supplementary_figures_9.jpeg]

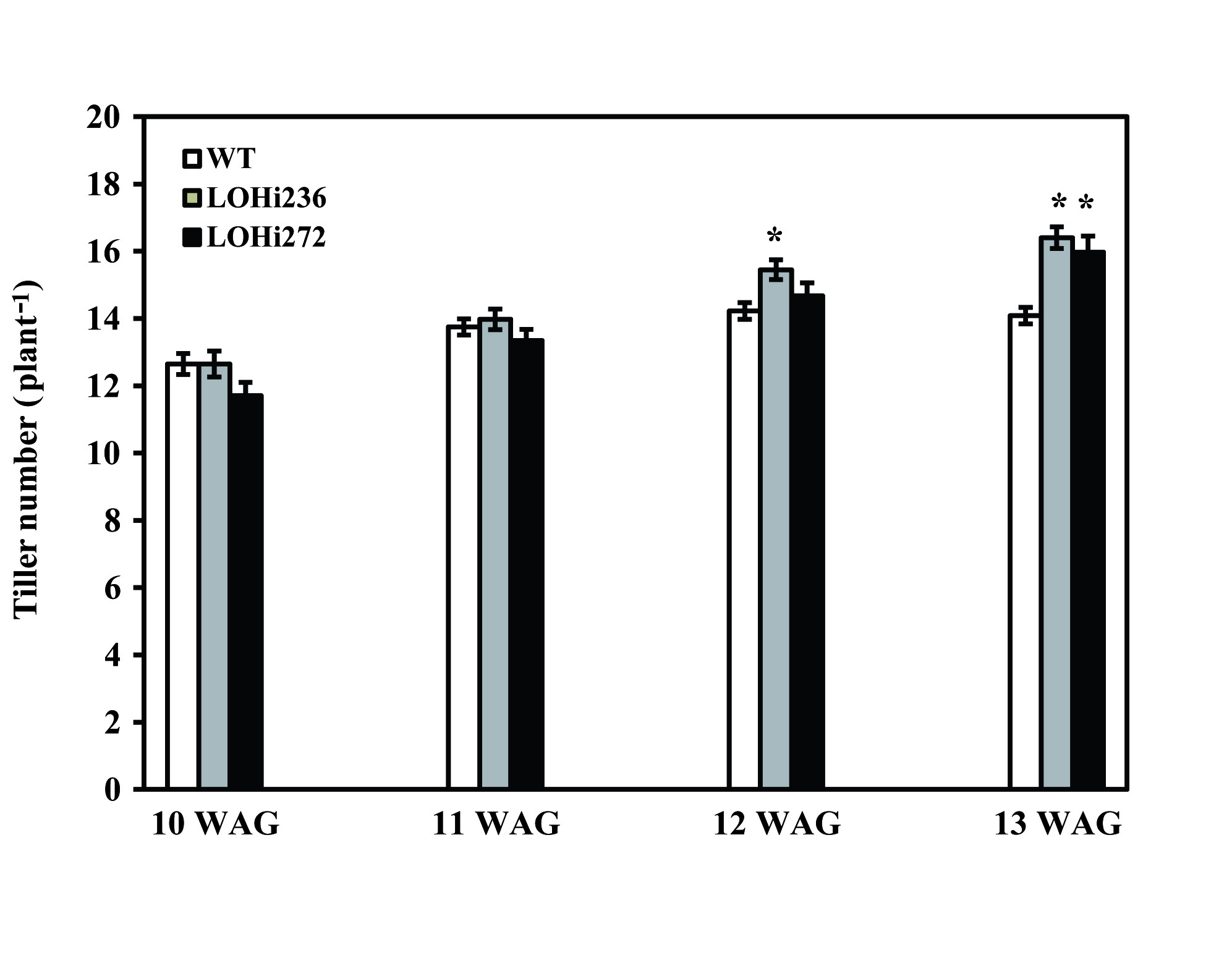

Supplement: Supplementary Figures 10 [file ery200_suppl_supplementary_figures_10.jpeg]

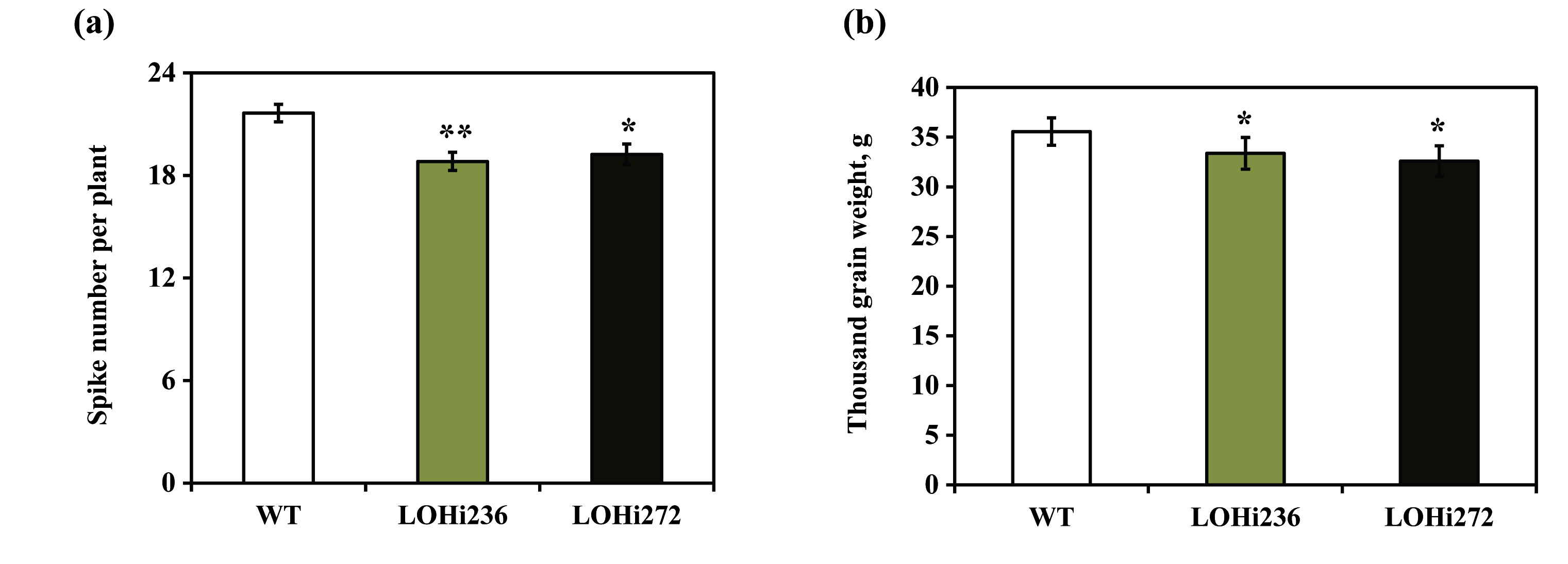

Supplement: Supplementary Figures 11 [file ery200_suppl_supplementary_figures_11.jpeg]

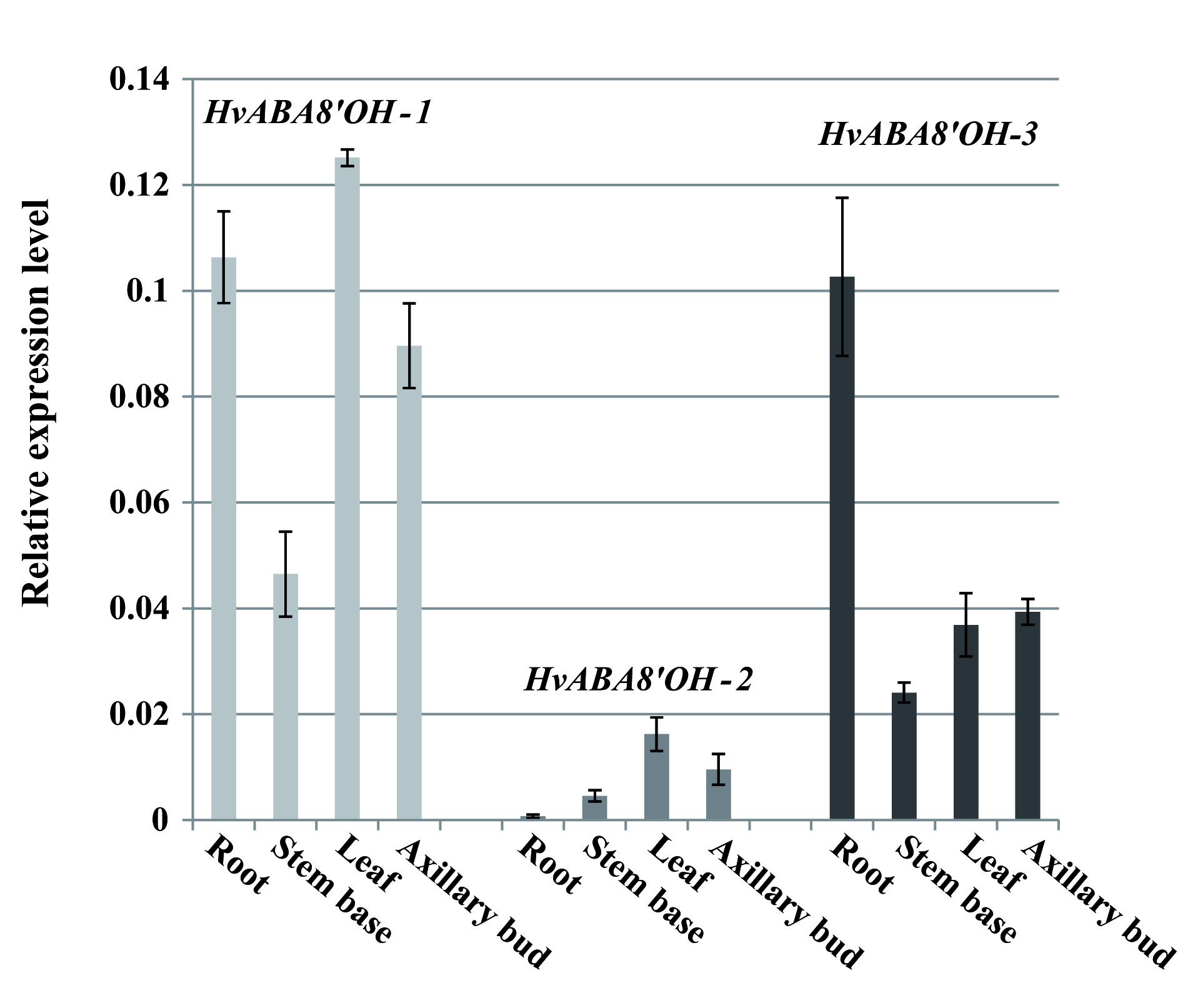

Supplement: Supplementary Figures 12 [file ery200_suppl_supplementary_figures_12.jpeg]

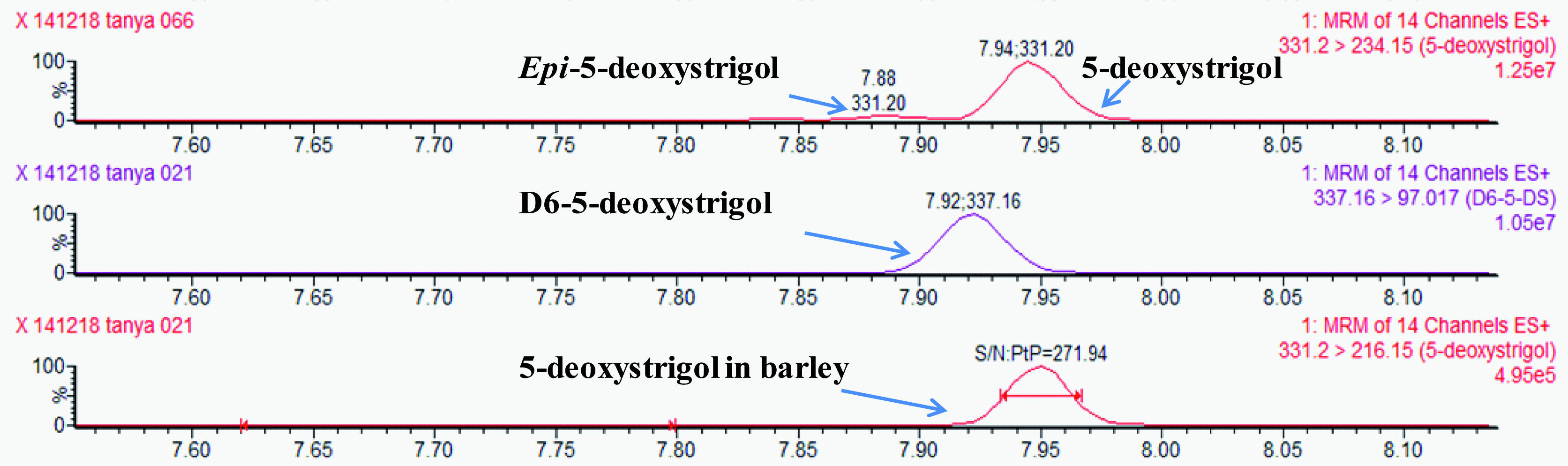

Supplement: Supplementary Figures 13 [file ery200_suppl_supplementary_figures_13.jpeg]

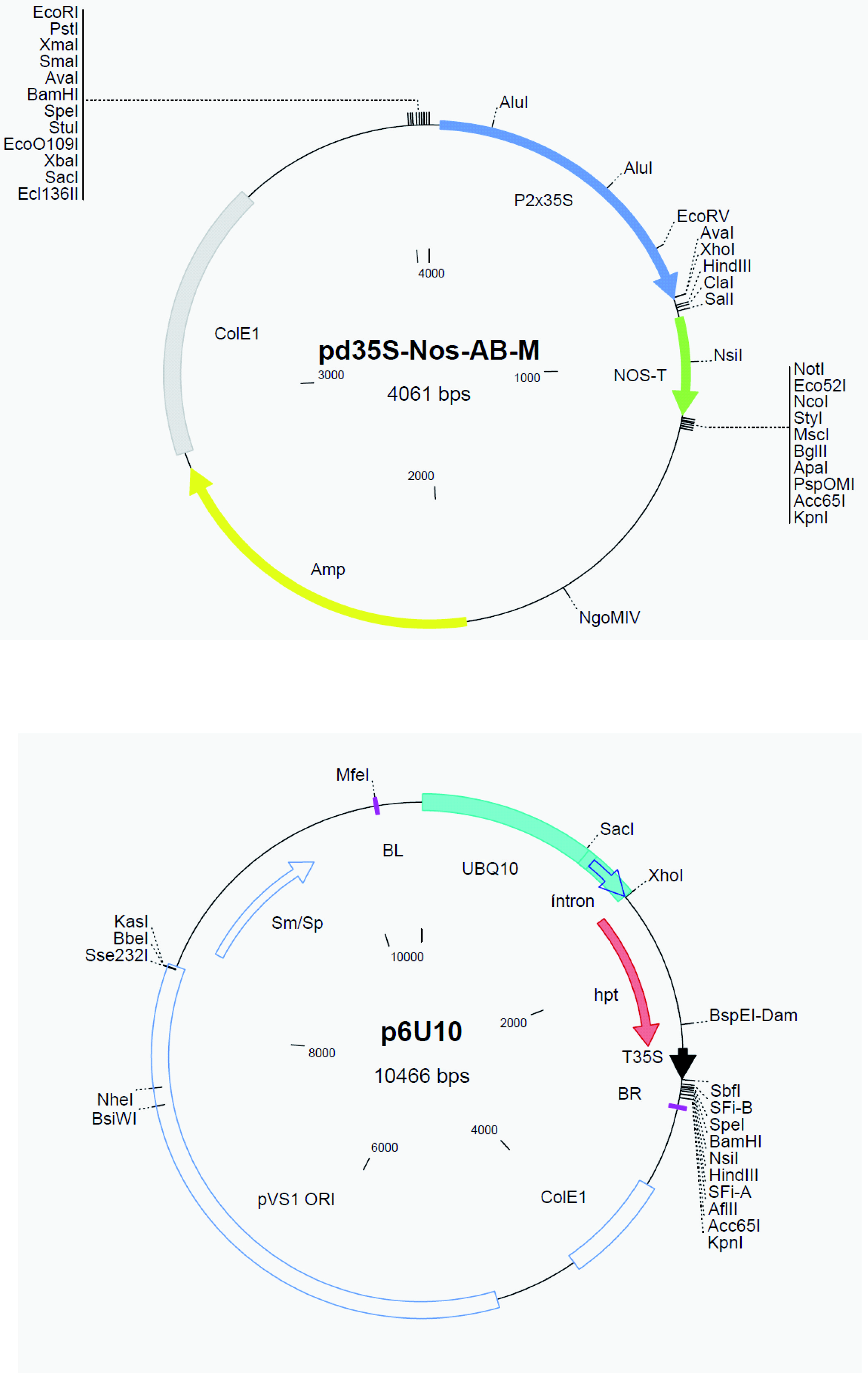

Supplement: Supplementary Figures 14 [file ery200_suppl_supplementary_figures_14.jpeg]
